# Printable Piezoresistive Hybrid Hydrogels Based on 2D MoS_2_ for Flexible Pulse Sensing

**DOI:** 10.1002/advs.77092

**Published:** 2026-08-27

**Authors:** Jenny Flores, Arianna Bertero, Luca Ceseracciu, Daniel Jiménez‐Boland, Sara Domenici, Mattia Bramini, Paola Sánchez‐Moreno, Erica Scarcelli, Domenico Galdiero, Pavel Fedorov, Daniil Karnaushenko, Rossella Sesia, Ignazio Roppolo, Micaela Pozzati, Mattia Spedicati, Nicolò Petrini, Marco Sangermano, Bartolomeo Coppola, Teresa Gatti

**Affiliations:** ^1^ Department of Applied Science and Technology Politecnico di Torino Torino Italy; ^2^ Materials Characterization Facility Istituto Italiano di Tecnologia Genova Italy; ^3^ Department of Cell Biology Faculty of Sciences Universidad de Granada Granada Spain; ^4^ Department of Applied Physics Faculty of Sciences Universidad de Granada Granada Spain; ^5^ Center for Sustainable Future Technologies (CSFT) Italian Institute of Technology (IIT) Torino Italy; ^6^ Dropper S.r.l. Torino Italy; ^7^ Research Center for Materials, Architectures and Integration of Nanomembranes Chemnitz University of Technology Chemnitz Germany; ^8^ Department of Mechanical and Aerospace Engineering Politecnico di Torino Torino Italy

**Keywords:** 2D material, chitosan, digital light processing 3D printing, heart rate sensor, hybrid hydrogel

## Abstract

The incorporation of two‐dimensional (2D) nanomaterials such as molybdenum disulfide (MoS_2_) nanoflakes/nanosheets into hydrogel matrices offers significant potential for the development of flexible and biocompatible piezoresistive sensors. Scalable synthesis methods, including liquid‐phase exfoliation and hydrothermal processes, enable the efficient production of 2D MoS_2_while reducing cost and environmental impact. Owing to the high surface area, anisotropy, and tunable electronic properties, these materials enhance sensitivity, mechanical strength, and electrical performance in hybrid systems. In this work, 1T‐ MoS_2_‐based hybrid hydrogels were fabricated via UV curing by forming cross‐linked networks with polyethylene glycol diacrylate (PEGDA) and chitosan (CH). Electrical conductivity was further improved through in situ interfacial polymerization of aniline. The equivalent 3D‐printed hydrogels exhibited enhanced mechanical strength, few‐cycles structural stability, swelling, and controlled deformation compared to simply UV‐cured systems, enabling more reproducible electromechanical responses. Both fabrication approaches showed linear and reversible piezoresistive behavior within the physiological blood pressure range. Furthermore, the optimized hydrogel demonstrated to be reliable in pulse monitoring (ca. 85 bpm) with good temporal correlation to the electrocardiogram signal and biocompatibility with human fibroblast cells, underscoring its potential for soft, flexible, and bio‐integrated sensing applications.

## Graphical abstract

Hybrid 1T‐MoS_2_‐based hydrogels are engineered through UV curing and 3D printing to achieve tunable electromechanical properties for wearable sensing. These soft architectures enhance mechanical stability and reproducible piezoresistive responses within the physiological pressure range. Reliable pulse monitoring, ECG correlation, and biocompatibility highlight their potential as next‐generation bio‐integrated sensors for cardiovascular monitoring.

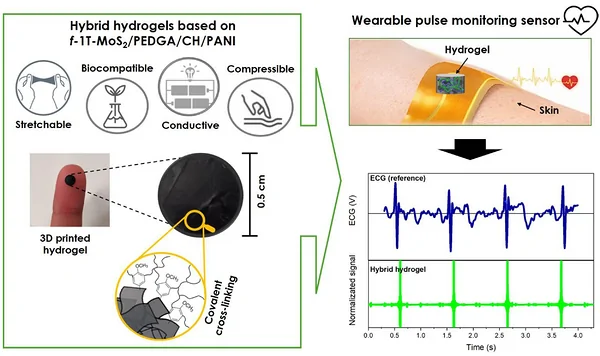

## 1 Introduction

With the aim of improving our quality of life and preventing health complications, the development of efficient wearable biomedical devices continues to be a trending topic in scientific research [1]. Among these, researchers are targeting devices for heart rate monitoring. Indeed, this is especially important since cardiovascular diseases, despite being largely preventable through early detection, remain a leading cause of mortality worldwide. Conventional modern diagnostic methods rely on devices that show various limitations in terms of portability and their ability to perform long‐term and continuous measurements. To support the diagnostic process and enable effective tracking of cardiovascular conditions, wearable sensing devices provide real‐time, non‐invasive monitoring [2, 3]. Among the various physiological parameters, blood pressure is a key indicator of cardiovascular health and is closely linked to heart rate dynamics [4]. Continuous monitoring of blood pressure, therefore, plays a vital role in early diagnosis and management of heart‐related conditions. However, the development of such devices comes with a series of challenges, such as ensuring comfort of wearability, biocompatibility of the employed materials, and good pressure sensitivity. Recent advances in flexible materials have enabled the design of wearable sensors that potentially meet these requirements [5]. Among these materials, hydrogels have emerged as promising scaffolds, offering tunable mechanical properties and excellent biocompatibility. Hydrogels are particularly attractive for wearable sensors due to their tissue‐like softness, high water content, and tunable mechanical and chemical properties, which allow them to conform intimately to the skin while maintaining their functionality [6]. To ensure the possibility of measuring an electric response to external pressure, the material must also be conductive; hence, the employment of conductive fillers that do not compromise the softness, wearability, and sensing properties of the materials is also crucial [7]. Nanofillers such as metal nanoparticles [8], carbon nanotubes (CNTs) [9], and two‐dimensional materials, such as MXenes [10], graphene oxide [11], and transition metal dichalcogenides (TMDs), are often employed to yield hybrid hydrogels [12].

However, each of these classes presents trade‐offs that can limit their application in soft, biointegrated sensing systems. Metal nanoparticles, while highly conductive, may suffer from aggregation, cytotoxicity concerns, and poor interfacial compatibility with polymer matrices [13].

MXenes have already been successfully used in wearable hydrogels, however their processing often requires exfoliation through harsh and dangerous conditions [14, 15]. Graphene oxide has also often been implemented in hydrogel sensors; nonetheless, its biocompatibility remains debated, showing dependence on dosage and synthetic procedures [16]. CNTs are highly hydrophobic, which makes their incorporation into hydrogels challenging and can introduce cytotoxicity issues [17, 18, 19]. Among TMDs, molybdenum disulfide (MoS_2_) emerges as an attractive choice due to its low toxicity, tunable electronic properties, and facile processability [20, 21].

Moreover, as a two‐dimensional material, MoS_2_ has an intrinsically anisotropic layered structure, which is particularly beneficial for strain‐sensing applications, as it can promote a more efficient coupling between mechanical deformation and electrical signal generation. Several studies have already proposed strategies for incorporating 2D MoS_2_ flakes into soft matrices, enabling piezoresistive hydrogels with self‐healing and high‐strain tolerance. For example, MoS_2_ can be functionalized to form covalent hybrid organic–inorganic networks. In particular, the 1T‐phase of MoS_2_ is highly electron‐rich, making it reactive toward electrophilic molecules, and it is also water‐dispersible, which makes it compatible with aqueous environments commonly used in hydrogel fabrication [22]. Moreover, the high electron density of the 1T‐phase is essential for translating mechanical pressure into measurable electrical signals. Taken together, these properties make 1T‐MoS_2_ a compelling choice for the development of soft, wearable piezoresistive sensors.

Despite the rapid development of conductive hydrogel‐based pressure sensors, several challenges remain. Existing systems typically focus on optimizing either the conductive filler, the polymer matrix, or the fabrication strategy individually. Comparatively few studies integrate sustainable 2D nanomaterial synthesis, architected additive manufacturing, and flexible device implementation within a single platform while preserving physiological sensing performance. Bridging these aspects is essential for translating advanced hydrogel materials into practical wearable sensing technologies. In a recent study carried out by our group, 1T‐MoS_2_ was prepared via chemical exfoliation, through lithium‐ion intercalation, followed by liquid‐phase exfoliation (LPE), producing 2D nanoflakes subsequently functionalized using aryl diazonium salts. A UV light‐triggered photopolymerization of poly(ethylene glycol) diacrylate (PEGDA) was then performed, yielding a covalently crosslinked hybrid hydrogel [23]. To enhance the mechanical stability and electrical conductivity, polyaniline (PANI) was incorporated into the hydrogel [23]. The final composite gel was tested for pressure sensing applications. In this work, we investigated alternative strategies to enhance sensor performance through a comparative study of hydrogels incorporating 1T‐MoS_2_ nanoflakes prepared via two distinct methods [24]: top‐down chemical exfoliation using n‐butyllithium (1T‐MoS_2‐_ *_exf_*) and bottom‐up hydrothermal synthesis using _L_‐Cysteine (1T‐MoS_2‐_ *_hyt_*). Chemically exfoliated 1T‐MoS_2_ often suffers from poor stability and involves hazardous, costly precursors that limit its scalability and commercial viability. Conversely, the hydrothermal synthesis route yields more stable materials and offers a more sustainable and scalable alternative [25]. This method benefits from low‐cost, non‐hazardous precursors and solvents, enhanced operational stability, and greater feasibility for large‐scale production. Moreover, to address the mechanical limitations of hydrogels in dynamic and mechanically demanding biological environments, a potential approach is the formulation of a double‐network hydrogel matrix [26]. Double‐network hydrogels are composed of two interpenetrating polymer networks, typically combining a rigid, brittle component with a soft, stretchable matrix. Herein, this was achieved by incorporating chitosan (CH) as an abundant, biocompatible filler, which further enhanced both the mechanical stability and sensitivity of the gels [27, 28].

To fabricate the hybrid hydrogels in this work, traditional techniques were used with silicone molds under a UV–vis lamp; however, this fabrication technique results in hydrogels with limited structures and shapes. For these reasons, the use of 3D printing technology was chosen as a powerful tool that allows the fabrication of reliable, reproducible, conductive hydrogels with specific structures and properties, in addition to being cost‐effective and easy to use [29, 30, 31, 32].

In particular, DLP additive manufacturing enables the fabrication of complex triply periodic minimal surface (TPMS) architectures with high geometrical precision, which are difficult to achieve using conventional molding approaches. TPMS geometries, such as gyroids, are especially attractive for electromechanical and wearable sensing applications because their continuous and smoothly interconnected surfaces promote homogeneous stress distribution, efficient load transfer, mechanical compliance, and interconnected conductive pathways [33, 34, 35, 36].

Therefore, this research has used digital light processing 3D printing (3D‐DLP) as a technique to create hydrogels with specific properties, dimensions, and geometries. Two types of geometries have been explored: (1) a dense cylinder and (2) a gyroid cylinder [37], with the aim of comparing their behavior as piezoresistive sensors and subsequently implementing them in a wearable device [38].

The gyroid architecture was selected as a representative TPMS structure capable of combining controlled porosity, structural continuity, and tunable deformation behavior, enabling modulation of the electromechanical response of the conductive hydrogel system. Overall, through these strategies, we have developed a hybrid hydrogel system that is both biocompatible, scalable, and suitable for continuous cardiovascular monitoring through piezoresistive response. 1T‐MoS_2‐_ *_exf_*‐based systems exhibit high sensitivity (GF 21.7), while 1T‐MoS_2‐_ *_hyt_*‐based ones provide enhanced mechanical compliance and stability. Chitosan incorporation improves energy dissipation, reducing hysteresis under cyclic loading. Using 3D‐DLP, architectures can be precisely controlled: dense structures achieve high pressure sensitivity (0.0337 kPa^−^^1^) and stable signals, whereas gyroid structures show higher strain sensitivity (GF 2.44). Finally, to demonstrate practical applicability, the optimized hydrogel was integrated into an ultrathin flexible sensing platform, enabling continuous pulse monitoring under wearable conditions. The optimized hydrogel enables pulse monitoring (ca. 85 bpm), with good electrocardiogram (ECG) correlation, and demonstrates biocompatibility, highlighting its potential for flexible and skin‐conformable wearable sensing applications.

## 2 Results and Discussion

### 2.1 Synthesis and Characterization of 1T‐MoS_2‐_ *_exf_* and 1T‐MoS_2‐_ *_hyt_*

**SCHEME 1:**
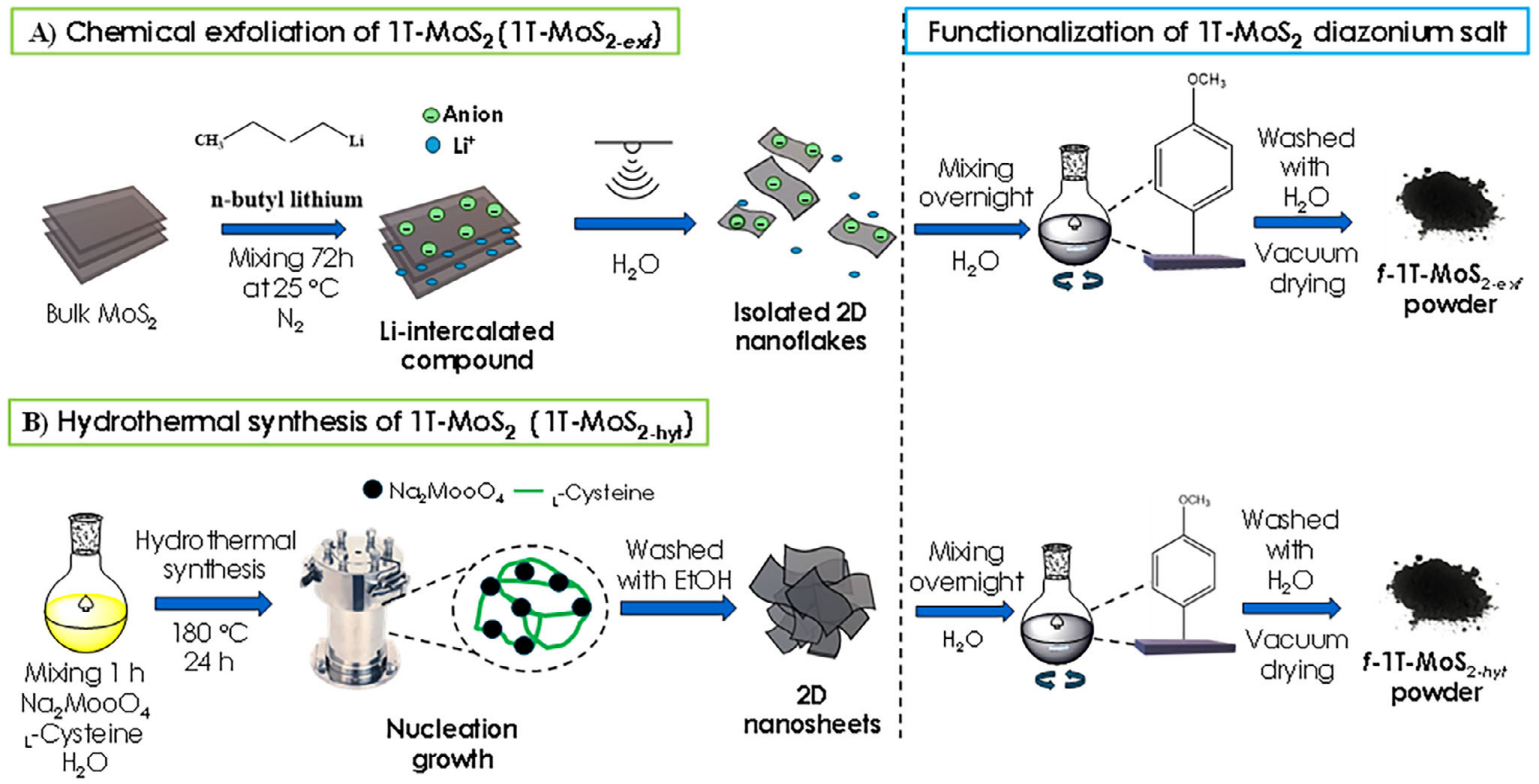
Synthesis and functionalization of 1T‐MoS_2‐_ *_exf_* nanoflakes (A) and (B) 1T‐MoS_2‐_ *_hyt_* nanosheets.

Nanostructured 2D 1T‐MoS_2_ was obtained using two different approaches: (A) chemical exfoliation of bulk MoS_2_ (top‐down approach) and (B) a one‐step green hydrothermal synthesis (bottom‐up approach). A detailed description of both procedures is provided in the Experimental Section, and their schematic representation is shown in Scheme 1.

Briefly, when working with the first method, the chemical exfoliation of bulk MoS_2_ is facilitated by solvated radicals generated from n‐butyllithium, which subsequently reacted with water during the exfoliation process via lithium‐ions intercalation, followed by a tip sonication in water to perform LPE [39]. This process increased interlayer distance due to Li ion intercalation and coulombic repulsion between the reduced MoS_2_ layers, resulting in the formation of nanoflakes primarily in the 1T‐MoS_2‐_ *_exf_* phase. For the second method, a modified one‐step hydrothermal method was used (Scheme 1B). For this synthesis, _L_‐Cysteine, a non‐toxic amino acid, was used as a sulfur precursor, reducing molybdenum from Mo^6+^ to Mo^4+^. After the 24 h hydrothermal reaction at 180°C, uniformly suspended 1T‐MoS_2‐_ *_hyt_* particles were obtained. After obtaining 1T‐MoS_2‐_ *_exf_* and 1T‐MoS_2‐_ *_hyt_* materials, the nanoflakes/nanosheets were chemically modified through a reaction with a diazonium salt (4‐methoxybenzenediazonium tetrafluoroborate) to form functionalized *f*‐1T‐MoS_2‐_ *_exf_* and *f*‐1T‐MoS_2‐_ *_hyt_* with pendant *p‐*methoxyphenyl substituents (─OCH_3_). All the details of the functionalization procedure are reported in Section S1.

The synthesized 1T‐MoS_2‐_ *_exf_* and 1T‐MoS_2‐_ *_hyt_* materials were characterized by transmission electron microscopy (TEM) to observe their morphology and nanostructure. For 1T‐MoS_2‐_ *_exf_*, nanoflakes (Figure S1) up to 500 nm wide and a few layers in thickness were observed (Figure S1B). For the 1T‐MoS_2‐_ *_hyt_*, individual sheets with sizes smaller than 100 nm were observed (Figure S1C), exhibiting randomly oriented, densely arranged, flower‐like morphologies with thin aggregated sheets (Figure S1D), as also described by Sesia et al.

Figure S2 shows the Zeta potential measurements of the 1T‐MoS_2‐_ *_exf_* and 1T‐MoS_2‐_ *_hyt_* materials in aqueous suspension, with values equal to −11.51 ± 0.43 and −26.45 ± 3.06 mV, respectively [40]. These results show that for both materials, the surface of nanoflakes is negatively charged, indicating that they are capable of forming highly stable aqueous suspensions. However, the significantly more negative zeta potential of 1T‐MoS_2‐_ *_hyt_* indicates enhanced electrostatic stabilization and therefore greater stability in aqueous suspension relative to 1T‐MoS_2‐_ *_exf_*. The crystallinity of the 1T‐MoS_2‐_ *_exf_* and 1T‐MoS_2‐_ *_hyt_* materials was studied using XRD; as presented in Figure S3, 1T‐MoS_2‐_ *_exf_* (black) exhibits the typical diffractogram of MoS_2_, with a higher diffraction peak at 14.8° (002) of bulk MoS_2_. In comparison, the 1T‐MoS_2‐_ *_hyt_* material shows a less intense diffraction peak (002), suggesting the existence of few layers MoS_2_ nanosheets with a decrease in the interlayer distance [41].

This lower intensity also indicates a reduced crystallinity and the presence of thinner/few‐layer nanosheets, features that are generally associated with improved compatibility and dispersion within polymeric matrices. Furthermore, two peaks are observed in both materials, at 32.88° (100) and 57.33° (110): these broad, weak peaks indicate that the resulting MoS_2_ materials have a very small crystal size [42].

Raman spectroscopy was also employed to identify the presence of the 1T‐MoS_2_ phase in the synthesized materials (Figure S4A). The Raman spectra of 1T‐MoS_2‐_ *_exf_* and 1T‐MoS_2‐_ *_hyt_* exhibit characteristic peaks associated with the 1T‐MoS_2_ phase at 150 cm^−1^ (J_1_), 225 cm^−1^ (J_2_), and 336 cm^−1^ (J_3_) [43]. Additionally, the spectra reveal three distinct peaks at 284 cm^−1^ (E^1^_1g_), 382 cm^−1^ (E^1^_2g_), and 408 cm^−1^ (A_1g_), which are indicative to the 1T/2H hybridized phase MoS_2_ [44]. The presence of these peaks suggests that the solvothermal (1T‐MoS_2‐_ *_exf_*) and hydrothermal (1T‐MoS_2‐_ *_hyt_*) processes induce a partial phase transition from the 2H to the 1T structure. However, the persistence of the 2H‐MoS_2_ peaks in both Raman spectra (violet and black) indicates that the two synthetics methods achieved less than 100% yield for the 1T‐phase due to the intrinsic higher stability of the 2H‐phase [44]. Figure S4A shows two more intense peaks in sample 1T‐MoS_2‐_ *_exf_* corresponding to the Raman active E^1^_2g_ and A_1g_ modes in bulk. In contrast, the hydrothermally synthesized 1T‐MoS_2‐_ *_hyt_* sample exhibited attenuation and broadening of these modes, indicative of increased structural disorder and potentially higher surface reactivity, which may promote improved interaction with the resin matrix and functional groups.

This effect is particularly evident when the excitation energy range is 1.88–2.81 eV (Raman spectra were collected in this work using λ_532nm_ = 2.33 eV) at room temperature, which has been extensively analyzed in previous works [45].

Subsequently, the covalent chemical functionalization of the nanosheets was carried out using an organic phenyl ring containing an electron donor (─OCH_3_) [46]. The functional groups provide increased stability for the 1T‐phase of MoS_2_ nanoflakes/nanosheets, inhibiting the conversion to the thermodynamically stable semiconductor state (2H), as previously demonstrated [47]. To verify that the functionalization of MoS_2_ leading to the formation of (*f*‐MoS_2_(─Ph─OCH_3_) was successfully obtained, Raman (Figure S4B), FT‐IR spectroscopy (Figure S4C), and thermogravimetric analyses (TGA, Figure S5), were used. Weight loss for all the samples was calculated by considering the decomposition of MoS_2_ at 650°C, following Equation S1 (see Section S1 for details) [39].

The Raman spectra presented in Figure S4B provide clear evidence of the structural and phase differences between exfoliated (1T‐MoS_2‐_ *_exf_*) and hydrothermally synthesized (1T‐MoS_2‐_ *_hyt_*) materials. Both spectra exhibit characteristic J_1,_ J_2_, and J_3_ peaks, located at 150, 225, and 336 cm^−^^1^, respectively, which are well‐established fingerprints of the metallic 1T‐phase of MoS_2_. In addition, the attenuation and broadening of the E^1^_2g_ and A_1g_ modes further support the reduced crystalline and increased structural disorder typically associated with hydrothermal synthesis. The *f*‐1T‐MoS_2_ *_hyt_* sample displays enhanced intensity of the J peaks, suggesting successful stabilization of the 1T‐phase through functionalization, preventing relaxation to the semiconducting 2H‐phase. This behavior indicates a more effective stabilization of the metallic 1T‐phase and a better retention of its functional properties, which may be advantageous for maintaining the electrical and photothermal performance of the material within photocurable ink [48, 49].

TGA was also used to estimate the functionalization degree (FD), the number of introduced functional group molecules per MoS_2_ unit. As shown in Figure S5, 1T‐MoS_2_ exhibited weight loss in the range of 200°C–380°C, attributed to the partial oxidation of MoS_2_ to MoO_3_ in air. The most significant mass loss (> 50 wt.%) in all samples corresponded to the sublimation of MoO_3_ occurring at 710°C (Figure S5). Additionally, a minor mass loss (≈10 wt.%) below 100°C was observed, likely due to the removal of adsorbed water, consistently with literature [50]. The FD values for *f*‐1T‐MoS_2_ *_‐exf_* and *f*‐1T‐MoS_2_ *_‐hyt_* are reported in Table S1. The exfoliated sample (*f*‐1T‐MoS_2 ‐_ *_exf_*) exhibits the highest weight loss (41.80%) and the lowest FD (1.60), corresponding to a high surface coverage of 4‐methoxyphenyl groups (─Ph─OCH_3_) per molecule of MoS_2_. In contrast, the hydrothermally synthesized sample (*f*‐1T‐MoS_2_ *_‐hyt_*) displays a lower functionalization density (FD = 2.0), likely due to competitive site blocking by residual organic contaminants, as evidenced by the substantial mass loss (22.15%) in the non‐functionalized control 1T‐MoS_2_ *_‐hyt_*. This lower functionalization density may also be beneficial by limiting excessive organic surface coverage, thereby maintaining greater exposure of the MoS_2_ surface and potentially reducing aggregation phenomena and viscosity increases within the photocurable ink [51].

The FT‐IR spectra (Figure S4C) confirm the presence of the functional group ─OCH_3_ and the phenyl ring in the functionalized materials (*f*‐1T‐MoS_2‐_ *_exf_* and *f*‐1T‐MoS_2‐_ *_hyt_*). Figure S4C shows the spectrum of the phenyl group (C_6_H_5_─) and a methoxy group (─OCH_3_) with absorption bands at symmetric 1458 cm^−1^ and asymmetric 1435 cm^−1^ stretches for ─OCH_3_ group, along with C_6_H_5_‐O 1291 cm^−1^ and 1250 cm^−1^ and C_6_H_5_‐OCH_3_ 1025 cm^−1^ stretches, as well as the O‐CH_3_ rocking mode 1174 cm^−1^ [39]. The polar interaction between the MoS_2_ and the phenyl ring has four characteristic modes, split into two quadrant deformations around 1550−1600 cm^−1^ and two semicircle deformations around 1450−1500 cm^−1^. The most energetic semicircular deformation around 1500 cm^−^^1^ only appears in the IR spectrum when the phenyl ring has an electron donating group, coinciding with the intense peak at 1488 cm^−^^1^ in the spectrum of C_6_H_5_OCH_3_, confirming the functionalization of the materials [39]. The band at 910 cm^−1^ is due to the S─S bond, and a vibration at 677 cm^−1^ is detected that can be assigned to a S─C stretching vibration [52]. The absorption at 1617, 1339, 1118, 890, and 639 cm^−1^ are attributed to the MoS_2_ [53, 54].

### 2.2 Preparation and Characterization of UV‐Cured and DLP‐Printed Hybrid Hydrogels

First, the hybrid hydrogels were prepared by UV lamp free radical polymerization using PEGDA and CH, as described in the Experimental Section. PEGDA and CH were used as chemical crosslinkers because both are hydrophilic in nature and have optimal biocompatibility. It has also been shown that integrating CH increases the swelling stability of the resulting crosslinked hydrogel [55]. The objective of combining these two polymers into our hybrid hydrogels was mainly to provide flexibility and stiffness, improving their mechanical stability without compromising their electromechanical properties [56].

**SCHEME 2:**
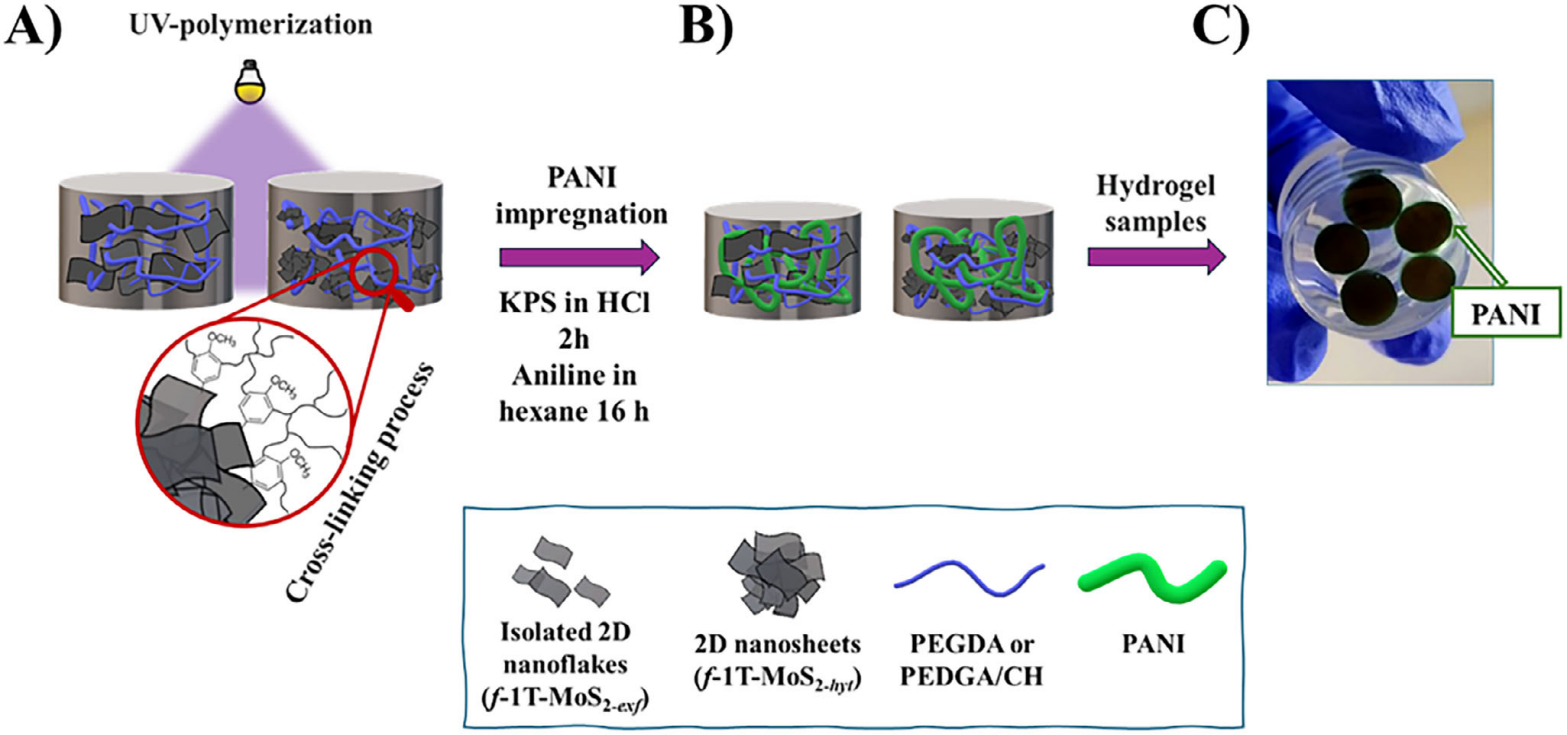
Schematic illustration of the preparation of the *f*‐1T‐MoS_2_ *_‐exf_* and *f*‐1T‐MoS_2_ *_‐hyt_*‐based PEGDA/PANI and PEGDA/CH/PANI hydrogels. (A) UV polymerization/crosslinking process to produce the hydrogels. (B) PANI impregnation into the hydrogels. (C) Real images of the UV‐cured hydrogels (size: ca.0.5 cm × 0.5 cm diameter × height).

Both *f*‐1T‐MoS_2‐_ *_exf_* and *f*‐1T‐MoS_2‐_ *_hyt_* were used to fabricate hybrid hydrogels. To do so, a mixture of PEGDA/CH and lithium phenyl‐2,4,6 trimethylbenzoylphosphinate (LAP) was prepared, and such a mixture was combined with the previously synthesized *f*‐1T‐MoS_2‐_ *_exf_* and *f*‐1T‐MoS_2‐_ *_hyt_* materials to undergo UV curing. This step (Scheme 2A) allowed for ionic crosslinking between the negatively charged 1T‐MoS_2_ flakes/nanosheets (through the pendant organic functional groups) and PEGDA/CH chains, resulting in hydrogel formation. Subsequently, DLP‐based 3D printing was performed using a simplified PEGDA‐based formulation without CH and incorporating *f*‐1T‐MoS_2‐_ *_hyt_* as the conductive phase.

The hydrothermally synthesized *f*‐1T‐MoS_2‐_ *_hyt_* material was selected for DLP processing due to its smaller nanosheet size, enhanced colloidal stability, lower crystallinity, and improved stabilization of the metallic 1T‐phase, all of which are advantageous for achieving homogeneous dispersion, reduced sedimentation, and minimized light scattering during photopolymerization. A simplified system was intentionally employed as a proof‐of‐formulation to evaluate the feasibility of the DLP process and the intrinsic piezoresistive performance of the PEGDA/*f*‐1T‐MoS_2‐_ *_hyt_* composite without additional compositional variables. The exclusion of CH from the DLP‐printable formulation was necessary at this preliminary stage, as its incorporation negatively affected resin viscosity, light penetration, and printing fidelity during the layer‐by‐layer photopolymerization process. Therefore, the simplified PEGDA‐based formulation was intentionally employed as a proof‐of‐formulation system to evaluate the feasibility of DLP fabrication and the intrinsic piezoresistive response of the conductive composite under optimized printing conditions. Future work will focus on the optimization of CH‐containing photocurable formulations for high‐resolution DLP manufacturing to allow a direct one‐to‐one comparison with the UV‐cured CH‐containing hydrogels. PEGDA was selected as the network‐forming polymer to ensure optimal printability and spatial resolution during DLP processing. The precursor solution containing PEGDA, LAP, and *f*‐1T‐MoS_2‐_ *_hyt_* was processed by spatially controlled photopolymerized to fabricate miniaturized dense cylindrical structures and gyroids for piezoresistive evaluation of each geometry. The printing parameters are detailed in the Experimental Section. The following discussions and comparisons between UV‐cured and DLP‐printed systems should be interpreted considering the combined influence of both the fabrication method and the hydrogel composition.

Finally, PANI was impregnated into both UV‐cured and DLP‐printed hydrogels through interfacial polymerization (Scheme 2B). This treatment resulted in uniformly dark hydrogels exhibiting a characteristic light emerald‐green color associated with PANI chains, while preserving the structural integrity and crosslinked polymer network of the hydrogels (Scheme 2C). As reported in previous works [57], the interfacial polymerization reaction begins in the organic phase between the hexane and the hydrogel (the internal aqueous phase of the gel) as detailed in the Experimental Section. The positively charged (protonated) aniline monomers move from the hexane solution into the hydrogel, where they polymerize and form PANI chains. Since the acid‐doped PANI is soluble in water, the chains only grew within the hydrogel. Additionally, Wei et al. and Chao et al. demonstrated that the immobilization of MoS_2_ nanosheets into hydrogels enhances their colloidal stability, which may lead to stabilization of the 1T‐phase, as previously discussed, thus further improving the mechanical properties of the hydrogel [58, 59].

Figure S6 displays the equilibrium swelling ratios measured for the hybrid hydrogel based on PEGDA and PEGDA/CH with *f*‐1T‐MoS_2‐_ *_exf_* and *f*‐1T‐MoS_2‐_ *_hyt_*. The equilibrium swelling ratio was stabilized after ca. 100 min for the MoS_2‐_ *_exf_*/PEGDA/PANI, MoS_2‐_ *_hyt_*/ PEGDA/CH/PANI, and MoS_2‐_ *_exf_* /PEGDA/CH/PANI samples without desorption, which may indicate stable crosslinking of the hydrogels; however, for the MoS_2‐_ *_hyt_*/PEGDA/PANI sample, stability was reached in ca. 800 min. Hydrogels composed of PEGDA/PANI show higher swelling, as is the case for MoS_2‐_ *_hyt_*, which is the highest among all variants, with about 70% swelling, followed by MoS_2‐_ *_exf_* (∼ 50%), suggesting stronger crosslinking due to the higher functionalization of the material as seen from TGA analyses (Table S1) [60].

For PEGDA/CH/PANI hydrogels, prepared both with *f*‐1T‐MoS_2‐_ *_exf_* and *f*‐1T‐MoS_2‐_ *_hyt_*, a lower equilibrium swelling of ca. 10% was observed due to the incorporation of CH that reduces the swelling and improves the mechanical [61, 62]. The improved mechanical properties stability induced by CH can also be appreciated from the results of the mechanical analyses reported in Table 1. PEGDA/CH/PANI hydrogels have swelling values ∼30%–40% higher than those of the hydrogels based on PEGDA/PANI since the CH with carboxylate (─COO^−^) groups in the hydrogel structure generate electrostatic repulsion leading to a well‐crosslinked polymeric network that can restrict water absorption into the hydrogel matrix [63], whereas PEGDA is highly hydrophilic due to its ether (─O─) groups which favor water absorption even when the hydrogel network is well cross‐linked [64].

**TABLE 1:** Gauge factor (GF) values of UV‐cured 1T‐MoS_2_ hydrogel composites under applied strain.

Sample	Strain	GF
*f*‐1T‐MoS_2_ *_‐exf_*/PEGDA/PANI	0%–5%	21.7
*f*‐1T‐MoS_2_ *_‐exf_*/PEGDA/CH/PANI	0%–5%	9.8
*f*‐1T‐MoS_2_ *_‐hyt_*/PEGDA/PANI	0%–5%	14.7
*f*‐1T‐MoS_2_ *_‐hyt_*/PEGDA/CH/PANI	0%–5%	9.1

All swelling values were determined using a mass‐based definition. Although PANI was incorporated in both systems, the DLP‐printed PEGDA/PANI hydrogels (Figure S7) exhibited substantially higher swelling (∼ 200%) compared to UV‐Cured PEGDA/PANI samples (∼ 45%–70%). This difference is attributed to variations in effective crosslink density and microstructure induced by induced by significantly different exposure energies by comparing UV‐cured and 3D printed samples. The additive DLP fabrication allows the employment of lower exposure energy (101 mJ/cm^2^ vs. 9000 mJ/cm^2^ for UV‐Cured hydrogels), manufacturing less reticulated and more water permeable structures. At the same time, the layer‐by‐layer photopolymerization network uniformity and geometrical resolution, thereby enhancing water penetration into the mesh size [65]. Additionally, the printed geometry provides increased surface accessibility and potential interlayer free volume, further facilitating swelling. In contrast, UV curing with a lamp promotes a less uniform network, and the need for higher curing energy drastically increases the reticulation degree, thus resulting in restricted solvent diffusion and limiting mass retention [66].

### 2.3 Electromechanical Characterization of the UV‐Cured Hybrid Hydrogels and Pulse Detection

The electromechanical performance of the UV‐Cured hybrid hydrogels was evaluated using a dynamometer coupled with a source meter, over 10 mechanical cycles varying from 0.05% (minimum) to 20% strain (maximum), as described in the Experimental Section. Deformation was applied at 2 mm/min displacement rate. The mechanical response, visible in the stress–strain curves, is accompanied by a corresponding variation in current output (see inset Figures S8A–C and S9A–C).

The loading‐unloading cycles show low mechanical hysteresis for both materials (*f*‐1T‐MoS_2_ *_‐exf_*/PEGDA/CH/PANI and *f*‐1T‐MoS_2_ *_‐exf_*/PEGDA/PANI), which is the typical behavior of soft elastomeric materials during stretching and releasing processes [67].

The low hysteresis in the *f*‐1T‐MoS_2‐_ *_exf_*/PEGDA/PANI and *f*‐1T‐MoS_2‐_ *_exf_*/PEGDA/CH/PANI hydrogels indicates low energy loss in the materials caused by water flow and energy dissipation from the polymer network. It should be noted that where the matrix‐filler cohesion is poor, the polymer networks can suffer irreversible structural damage during multiple loading and unloading processes. For example, small fractures can form within the hydrogel matrix, preventing it from recovering its original shape and increasing the effects of hysteresis, as can be observed in the *f*‐1T‐MoS_2‐_ *_hyt_*/PEGDA/CH/PANI and *f*‐1T‐MoS_2‐_ *_hyt_*/PEGDA/PANI hydrogels (see Section S2 for details in Figure S9A–C).

Similarly, the large hysteresis of *f*‐1T‐MoS_2‐_ *_hyt_*/PEGDA/CH/PANI and *f*‐1T‐MoS_2‐_ *_hyt_*/PEGDA/PANI hydrogels (Figure S9A–C) can be generated mainly by the incorporation of small agglomerated nanosheets of MoS_2_, loosely interacting with the matrix, which interfere with the optimal cross‐linking of the hydrogels, reducing their resistance to viscous deformation and, consequently, their piezoresistive properties (Figure S9B–D) [68].

It can be observed (Figure S8B) that the variations of resistance of the *f*‐1T‐MoS_2_ *_‐exf_*/PEGDA/CH/PANI hydrogel are reproducible and durable in the strain range of 5%–20% during 10 loading/unloading cycles. The peak current variation, shown in the inset of Figure S8B, confirms that the electrical signals of the *f*‐1T‐MoS_2_ *_‐exf_*/PEGDA/CH/PANI hydrogel are stable, with values around 2.5 × 10^−^^4^ A for 400 s. On the other hand, Figure S8D shows less stable behavior of *f‐*1T‐MoS_2_ *_‐exf_*/PEGDA/PANI, and it can be observed that during each load/unload compression cycle the electromechanical resistance decreases and hysteresis increases; at the same time, it can be observed that the current decreases in response to the deformation of the material (inset in Figure S8D) with an overall decrement from 3.1 × 10^−^^4^ to 2.1 × 10^−^^4^ A in 400 s. This phenomenon can be attributed to the weakly crosslinked dual network and the poor interaction between MoS_2_ nanoflakes and the chitosan in the polymer matrix. These results indicate that the increase in electromechanical hysteresis influences the alteration of conduction mechanisms, causing a clear decrease in current overtime [10].

Figure S9B–D shows how the energy dissipation capacity of *f*‐1T‐MoS_2‐_ *_hyt_*/PEGDA/CH/PANI hydrogels affects the electric current variation upon compression of the material. In this case, the current remains stable (2.0 × 10^−^^5^ A after the first two cycles) during more than 100 s, compared to that of the *f*‐1T‐MoS_2‐_ *_hyt_*/PEGDA/PANI material (from 3.0 × 10^−^^4^ to 8.0 × 10^−^^5^ A), which is not stable in the first 120 s, and continues to decrease during each compression cycle of the hydrogel. This behavior is attributed to its lower energy dissipation capacity and inferior mechanical stability compared to the PEGDA/CH‐based network, leading to cumulative structural damage during cyclic testing [69].

Hydrogels made of pure PEGDA typically resist ultrafast and continuous cyclic compression, but fractures can generate in the hydrogel network structure, preventing the materials from returning to their original position; the reduction in the contact area between the agglomerates of the nanosheets consequently leads to an inferior current generation [10, 70]. The dissipation of charge energy in *f*‐1T‐MoS_2‐_ *_hyt_*/PEGDA/CH/PANI (Figure S9D) is mediated by dynamic supramolecular interactions between the methoxy groups of *f*‐1T‐MoS_2_ and the polymer matrix base on PEGDA/CH/PANI, as well as by the interfacial displacement of the nanosheets, which reorganize in a more orderly manner with a smooth increase in the contact area between the nanosheets. These phenomena may help reduce the fracture of the hydrogel network structures, thereby improving its mechanical properties.

The sensitivity of a strain sensor is defined as the gauge factor (GF), which was calculated as detailed in the Experimental Section. Figure S8B shows stable electromechanical performance characterized by an initial increase followed by saturation to a plateau. Table 1 shows the calculated GF values within the strain range of 0%–5%, corresponding to the linear regime before the plateau, and gave values of 21.7 for *f*‐1T‐MoS_2‐_ *_exf_*/PEGDA/PANI and 9.8 for *f*‐1T‐MoS_2‐_ *_exf_* /PEGDA/CH/PANI. GF for *f*‐1T‐MoS_2‐_ *_hyt_* /PEGDA/PANI was 14.7, and for *f*‐1T‐MoS_2‐_ *_hyt_* PEGDA/CH/PANI was 9.1. Interestingly, the addition of CH reduced the GF for both *f*‐1T‐MoS_2‐_ *_exf_* and *f*‐1T‐MoS_2‐_ *_hyt_*, which could be attributed to the finer initial percolation network present.

The *I–V* curves in Figures S10A and S12B show capacitive behavior with significant voltage displacement at null current, mainly attributable to the intrinsic pseudocapacitive properties of PANI. The properties of PANI with the *f‐*1T‐MoS_2‐_ *_exf_* and *f‐*1T‐MoS_2‐_ *_hyt_* hydrogels based on PEGDA/CH/PANI and PEGDA/CH/PANI, respectively, showed a behavior similar to that of a Schottky‐like behavior as previously described by Domenici et al. and Dai et al. [23, 71].

The resistances in Figures S10 and S11 were calculated from the *I–V* curves by applying Ohm's law. For all samples with the *f‐*1T‐MoS_2‐_ *_exf_* and *f‐*1T‐MoS_2‐_ *_hyt_* based on PEGDA/CH/PANI and PEGDA/PANI, the fitting was performed in the positive bias linear region. Quasi‐static compression tests showed that the PEGDA/PANI and PEGDA/CH/PANI‐based hydrogels (Figures S10 and S11C,D) displayed a decrease in resistance with increasing pressure when incorporating the *f‐*1T‐MoS_2‐_ *_exf_* and *f‐*1T‐MoS_2‐_ *_hyt_* materials into both matrices, indicating the ability to distinguish stresses of different pressure intensities (from 0.3 to *ca*. 2.3 kPa). The hydrogels *f‐*1T‐MoS_2‐_ *_exf_* and *f‐*1T‐MoS_2‐_ *_hyt_* based on PEGDA/CH/PANI showed similar resistance behaviors (Figure S10C,D), with no significant changes after 0.5 kPa of pressure, remaining at ca. 4000 MΩ and ca. 2500 MΩ for the *f‐*1T‐MoS_2‐_ *_exf_* and *f‐*1T‐MoS_2‐_ *_hyt_*, respectively.

In Figure S11C,D, it can be observed that the hydrogel with *f*‐1T‐MoS_2‐_ *_exf_* /PEGDA/PANI shows a low resistance after 0.5 kPa pressure (ca. 1100 MΩ), whereas the hydrogel with *f*‐1T‐MoS_2‐_ *_hyt_* /PEGDA/PANI shows a consecutive decrease in resistance with increasing pressure from 0.3 to 1.7 kPa with values ranging from ca. 1600 to 1100 MΩ, respectively. This behavior is attributed to the anisotropic effect provided by the 2D nanoflakes/nanosheets in the polymeric matrix, which causes these materials to distribute in a flat and organized manner within the matrix with increasing pressure, reducing the average distance between them and giving rise to a clear variation in resistance under compression [10, 72].

The superior compliance and higher GF of 1T‐MoS_2_ *_‐hyt_*‐based hydrogels within the physiological pressure range compared to the stiffer 1T‐MoS_2_ *_‐exf_* hydrogels. This favorable balance between mechanical adaptability and sensing performance motivated their selection for subsequent 3D printing and device fabrication.

**FIGURE 1:**
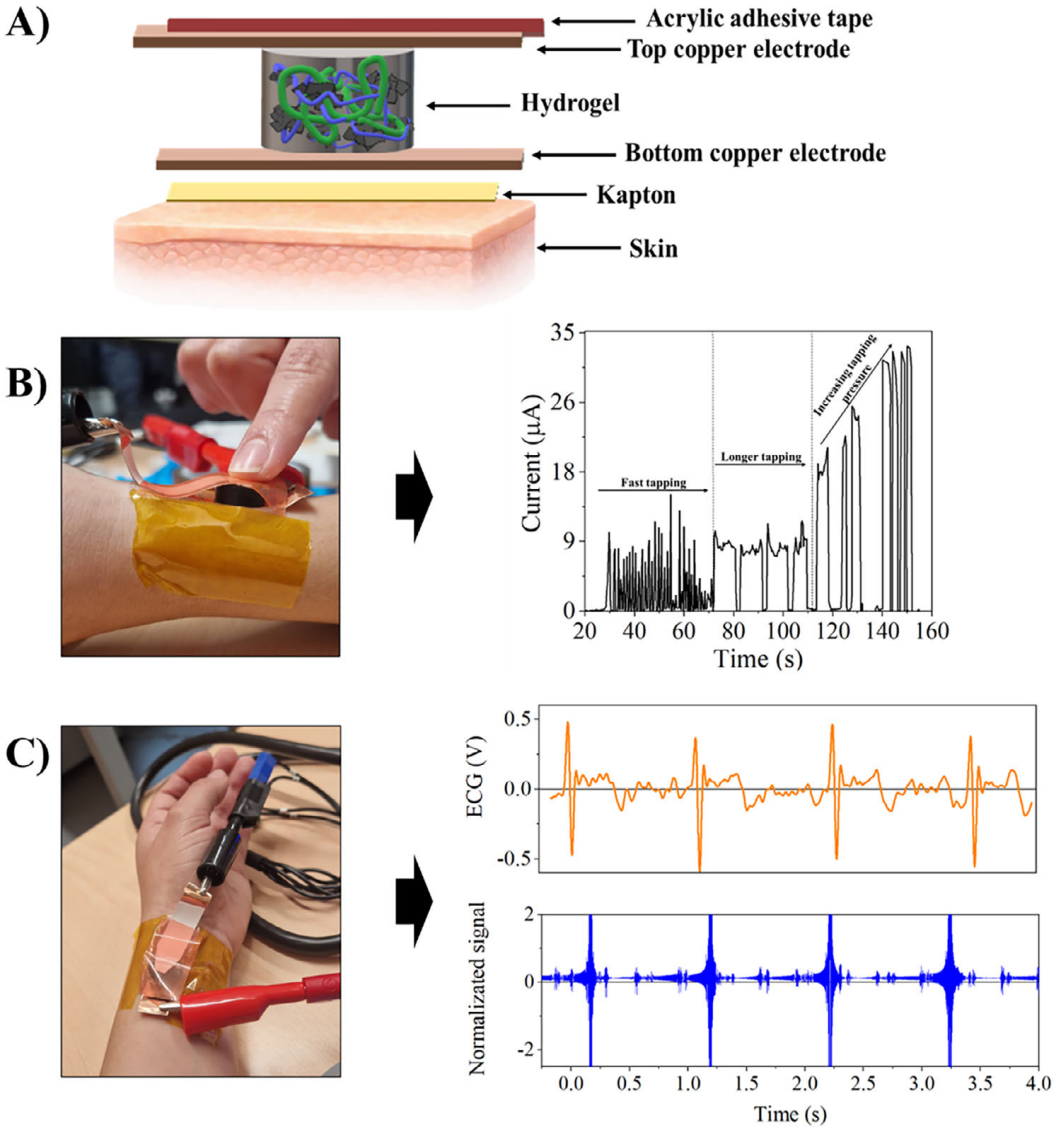
(A) Schematic representation of *f‐*1T‐MoS_2_ *_‐hyt_*/PEGDA/CH/PANI hydrogel measuring setup (left) and its chronoamperometric current response. Inset: picture of experimental setup. (B) Picture of the experimental setup used for the finger‐induced tapping (left) and the resulting chronoamperometric response. (C) Picture of the experimental setup used for the measurement of the resting pulse located on the wrist (left), the resulting ECG waveform (orange line), and the ultraprocessed normalized signal obtained from the hydrogel (blue line).

Figure 1 shows the chronoamperometric response of the *f*‐1T‐MoS_2‐_ *_hyt_*/PEGDA/CH/PANI hydrogel under dynamic mechanical stimulation and the pulse response measured on the wrist. Rapid and good sensitivity were observed during fast tapping (Figure 1B), demonstrating a fast electromechanical response and minimal hysteresis [73]. Under sustained pressure (Figure 1B), the current remains elevated, indicating reduced inter‐sheet separation and enhanced electron tunneling between adjacent 1T‐MoS_2‐_ *_hyt_* nanosheets mediated by the PANI matrix. Moreover, increasing applied pressure results in a corresponding rise in peak current, confirming the pressure‐dependent conductivity of the composite. This behavior is attributed to compression‐induced reduction in inter‐sheet spacing and improved electrical contact within the 1T‐MoS_2‐_ *_hyt_* network dispersed throughout the elastic PEGDA/CH matrix. The reversible current response further demonstrates the mechanical robustness and structural stability of the hybrid hydrogel, supporting its suitability for real‐time biomechanical sensing applications [23].

Figure 1C shows an example of the sensor prototype (Figure 1A), with which we detected the human pulse by placing the *f*‐1T‐MoS_2‐_ *_hyt_*/PEGDA/CH/PANI hydrogel over the radial artery of the wrist. The configuration was designed to evaluate the electromechanical response of the hydrogel under wearable operating conditions. Future developments will focus on miniaturized flexible electronics and wireless integration for fully wearable implementations. As shown in Figure S13, the average pulse frequency extracted from peak‐to‐peak intervals over 30 s was ca. 1.42 Hz, corresponding to ca. 85 beats per minute (bpm), calculated using to the formula *f* = 1/T, where *f* is the frequency, and T is the response time from the peak‐to‐peak intervals. This value falls within the typical resting heart rate range for healthy adults (60–100 bpm), demonstrating the sensor's capability for stable and accurate biomechanical signal detection [74, 75].

As shown in Figure 1C, the extracted pulse waveform is consistent with the cardiac activity observed in the simultaneously recorded ECG leads, demonstrating temporal synchronization between the hydrogel‐derived signal and the ECG waveform. This correspondence confirms the sensor's capability for real‐time cardiovascular monitoring. Recent reviews [75, 76] detail the use of flexible piezoresistive pressure sensors, which have been extensively studied for non‐invasive pulse waveform monitoring due to their simple mechanical‐electrical transduction and their suitability for flexible integration and adaptation to the skin. In this regard, the slight delay observed for the signal taken from the hydrogel compared to the ECG signal is likely the pulse transit time (PTT), a well‐known indicator for hypertension detection. The better understanding of this aspect remains anyway subject of future work [77, 78].

### 2.4 Cell Viability of MRC5 on the Hybrid Hydrogels

**FIGURE 2:**
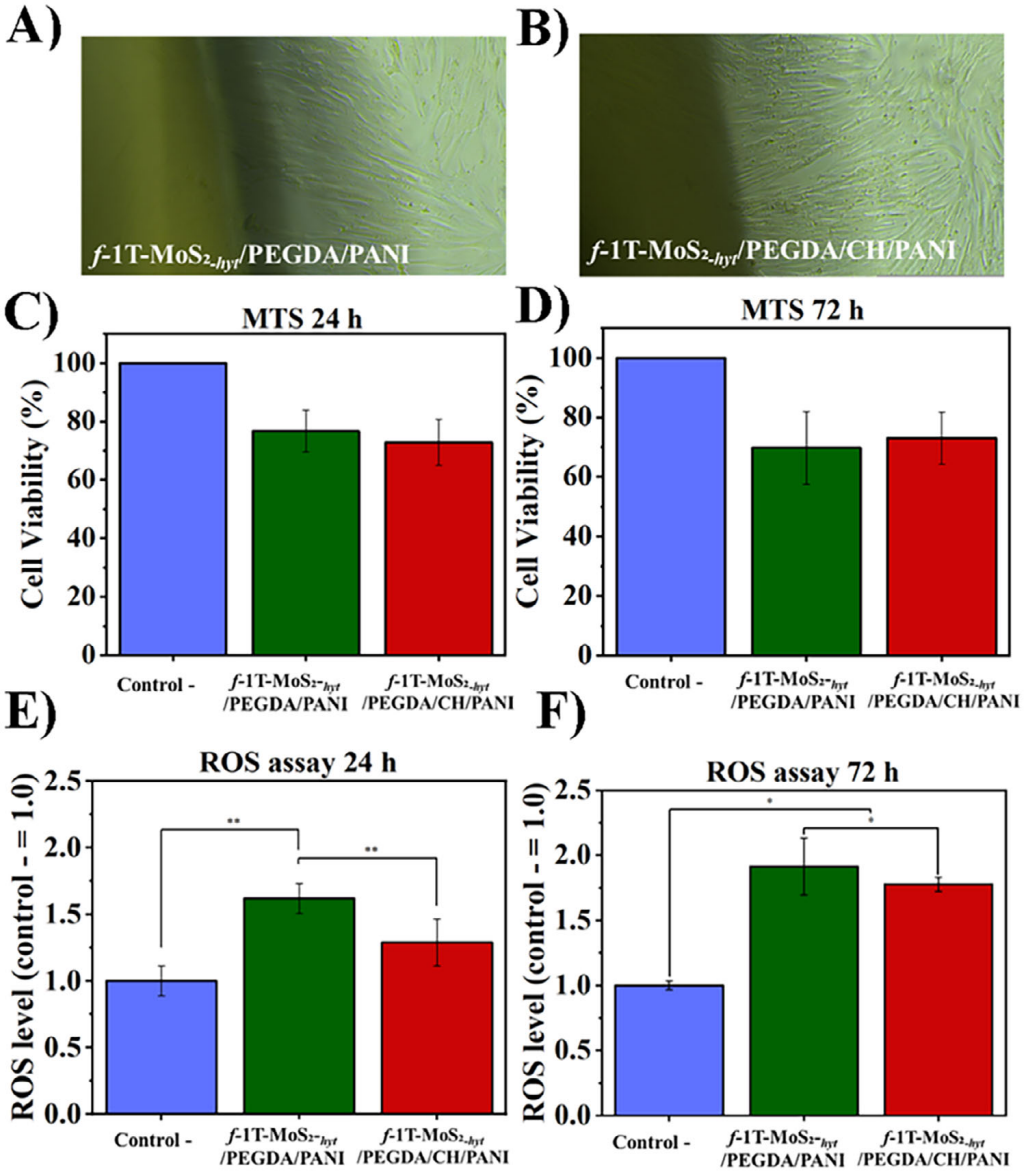
Biocompatibility of *f‐*1T‐MoS_2‐_ *_hyt_*/PEGDA/PANI and *f‐*1T‐MoS_2‐_ *_hyt_*/PEGDA/CH/PANI hydrogels with human fibroblast cells (MRC5 cell line). Micrographs of (A) *f‐*1T‐MoS_2_ *_‐hyt_*/PEGDA/PANI and (B) *f‐*1T‐MoS_2‐_ *_hyt_*/PEGDA/CH/PANI bio‐interactions with MRC5 cells under optical microscopy. Cell viability MTS assay (C, D) and ROS assay (E, F) of MCR5 cells with *f‐*1T‐MoS_2‐_ *_hyt_*/PEGDA/PANI and *f‐*1T‐MoS_2‐_ *_hyt_*/PEGDA/CH/PANI hydrogels in culture for 24 (C, E) and 72 h (D, F) of incubation. Statistically significant differences (one‐way ANOVA with Tukey B post‐hoc analysis, *p* < 0.05) are highlighted with “*” (C–F). The standard error (SEM) is displayed (three independent replicates).

Biocompatibility of *f*‐1T‐MoS_2‐_ *_hyt_*/PEGDA/PANI and *f*‐1T‐MoS_2‐_ *_hyt_*/PEGDA/CH/PANI hydrogels was assessed in human fibroblast cells (MRC5 cell line). Hydrogels were deposited on the surface of the MRC5‐cell monolayer for 24 and 72 h of incubation in order to evaluate the biological interaction of hydrogels with human fibroblasts (Figure 2A,B). Then, cell viability and oxidative state of MRC5 cells were evaluated using the CellTiter 96 AQueous One Solution Cell Proliferation Assay (MTS) (Promega) and the ROS‐Glo H_2_O_2_ Assay (Promega), respectively. Although the MRC5 incubation with both hydrogels apparently led to a slight decrease in cell viability, data did not show statistical significative differences compared to control cells (Control ‐) for 24 and 72 h of incubation (Figure 2C,D). Additionally, the ROS assay revealed a slight increase in ROS level of MRC5 cells subjected to hydrogels for 24 h, which was maintained over time for 72 h of incubation (Figure 2E–F). Nevertheless, the ROS level increase (1.29 ± 0.17) found in *f*‐1T‐MoS_2‐_ *_hyt_*/PEGDA/CH/PANI for 24 h of incubation (Figure 2E) was not found statistically significant compared to negative control cells (Control ‐).

Although further validation in other in vitro human skin cell models is required, the in vitro monolayer culture of MRC5 cells provides a preliminary indication of hydrogel biocompatibility with human fibroblasts. Accordingly, these results demonstrate the good biocompatibility of *f*‐1T‐MoS_2‐_ *_hyt_*/PEGDA/PANI and *f*‐1T‐MoS_2‐_ *_hyt_*/PEGDA/CH/PANI hydrogels, showing promise for translational research.

### 2.5 Electromechanical Characterization of the DLP‐Printed Hybrid Hydrogels

**FIGURE 3:**
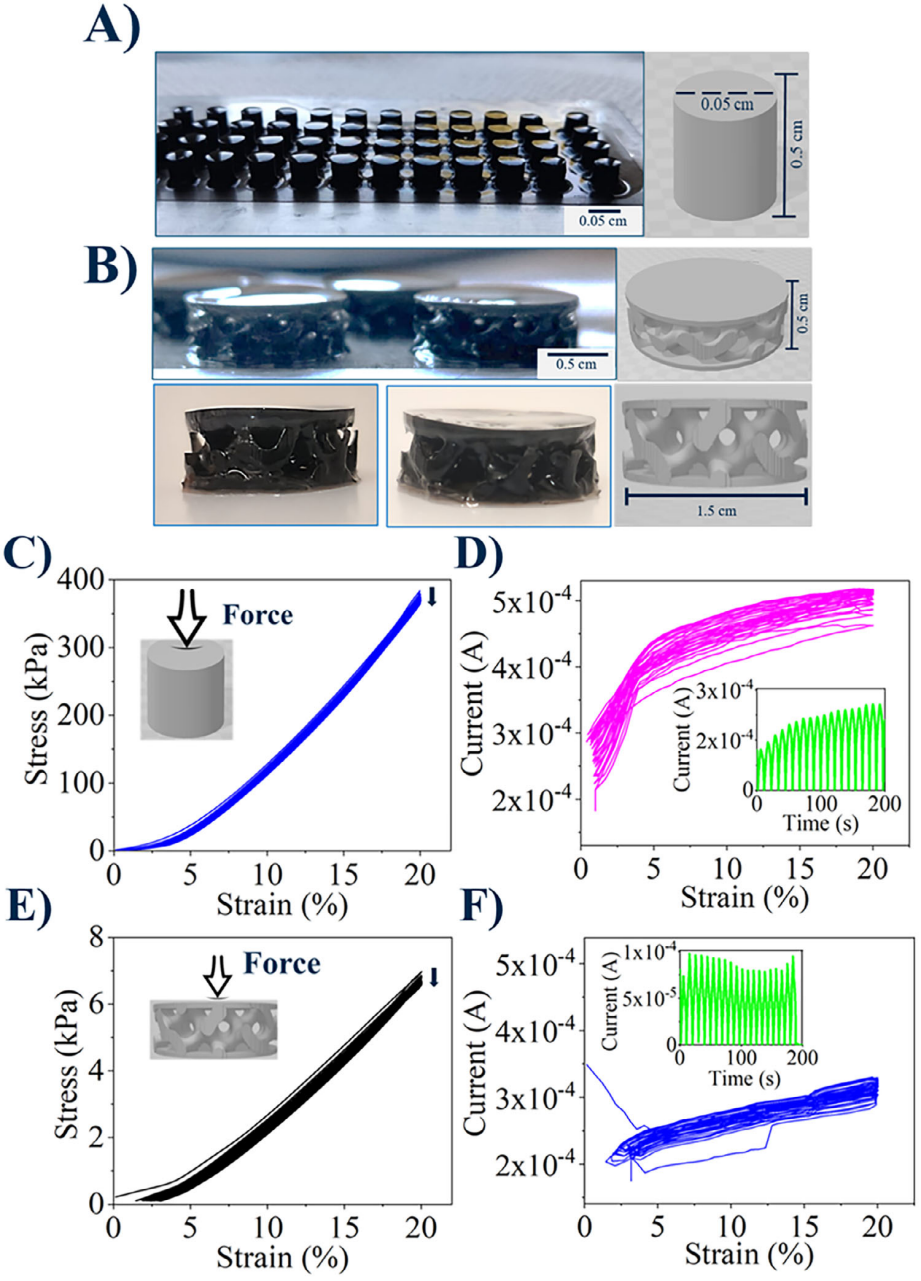
Digital photographs and designed models of *f*‐1T‐MoS_2‐_ *_hyt_*/PEGDA hydrogels: (A) Dense cylinders (diameter 0.5 cm, 0.5 cm height) and (B) Gyroid cylinders with up and down flat surfaces (diameter 1.5 cm, 0.5 cm height). Cyclic stress–strain curves and electromechanical response of 3D‐printed *f*‐1T‐MoS_2_ *_‐hyt_*/PEGDA/PANI piezoresistive hybrid hydrogel with: (C, D) Dense cylindric and (E, F) Gyroid cylindrical geometries [37]. Insets: show the electrical output performance of hydrogels for 20 cycles of cyclic pressure.

Figure 3 highlights the influence of structural architecture on the modulation of both mechanical compliance and sensing performance of 3D‐printed *f*‐1T‐MoS_2‐_ *_hyt_* /PEGDA/PANI hydrogels for wearable applications. To compare the architectural design (dense vs. gyroid, Figure 3A,B) on electromechanical performance, a single, well‐controlled material system was used for all DLP prints. Hydrothermally synthesized (*f‐*1T‐MoS_2‐_ *_hyt_*) for DLP processing due to its ease of reproducibility, smaller nanosheet size, enhanced colloidal stability, lower crystallinity, and improved stabilization of the metallic 1T‐phase, all of which are advantageous for achieving homogeneous dispersion, reduced sedimentation, minimized light scattering, and uniform curing during photopolymerization, ultimately enabling high‐fidelity fabrication by DLP. PEGDA served as the model for a photocurable matrix due to its established printability and predictable crosslinking behavior, allowing for systematic analysis of structure, properties, and performance without formulation‐related confounding effects. Particular attention was devoted to the fabrication of TPMS‐based gyroid architectures, since these structures are increasingly recognized as highly effective platforms for electromechanical and piezoresistive applications due to their smooth continuous surfaces, homogeneous stress redistribution, interconnected porosity, and bending‐dominated deformation mechanisms. The high geometrical freedom and spatial resolution offered by DLP printing were essential for accurately reproducing these complex architectures, which would be difficult to fabricate using conventional hydrogel processing methods. In the future, this structure will be extended to biodegradable systems (such as UV‐cured chitosan‐based hydrogel formulations, see the Experimental Section), based on the architecture‐response relationships established here.

The electromechanical performance of the 3D‐printed hydrogels was assessed over 20 cycles (0.05%–20% strain, as described in the Experimental Section) at a deformation rate of 2 mm min^−^^1^. The number of cycles was chosen as a compromise between long‐term robustness assessment and the practical feasibility of testing a large set of samples. A comprehensive durability evaluation is deferred to future work. The stress–strain response was correlated with changes in current output (see inset Figure 3D–F). The dense cylindrical geometry exhibits higher compressive stiffness and uniform deformation, resulting in stable and reproducible electromechanical behavior under cyclic loading (Figure 3C–F). Quantitatively, the dense structure presents a strain gauge factor (GF) of 1.06 and a pressure sensitivity of 0.03371 (ΔR/R_0_ kPa^−^^1^, Figure 3C), shows stable and reproducible current modulation under cyclic compression (Figure 3D), with minimal hysteresis and negligible baseline drift, reflecting consistent conductive pathway evolution within the *f‐*1T‐MoS_2_ *_‐hyt_*/PANI network, indicating reliable resistance modulation both in terms of strain and compressive stress (Figure 3C). Notably, the strain detection range explored in this study falls within the physiological blood pressure window (≈10–16 kPa, corresponding to a diastolic pressure of 80–120 mmHg and a systolic pressure of 80–120 mmHg), highlighting the system's suitability for biomechanical sensing applications. In contrast, the gyroid architecture demonstrates enhanced strain sensitivity, with a GF of 2.44, but significantly lower pressure sensitivity (0.00432 ΔR/R_0_ kPa^−^^1^, Figure 3E), which dynamically alters the contact between 1T‐MoS_2_ *_‐hyt_* nanosheets and the density of the hydrogel's conductive junctions (Figure 3F).

Additionally, the higher porosity of the gyroid architecture may facilitate internal water redistribution under load, further contributing to signal fluctuations. This increased strain responsiveness arises from bending‐dominated deformation of the thin struts, which amplifies local strain within the conductive network [79, 80]. The continuous TPMS geometry of the gyroid structure also promotes stress redistribution and interconnected conductive pathways throughout the hydrogel volume, features that are considered highly advantageous for flexible electromechanical systems and wearable sensing platforms. However, the heterogeneous deformation and dynamic reconfiguration of conductive pathways in the porous gyroid structure also lead to greater signal dispersion and reduced stability under compressive loading [81]. From a wearable sensing perspective, the dense configuration offers superior pressure detection capability and signal stability compared to UV‐cured hydrogels (Table 1), which, although they exhibit higher intrinsic sensitivity (GF of up to 21.7), exhibit increased hysteresis and reduced signal reproducibility under cyclic loading due to their less controlled architecture and limited structural integrity. In contrast, the 3D‐printed dense hydrogels enable more uniform stress distribution and stable conductive pathway evolution, resulting in improved signal consistency. While the gyroid structure provides higher strain sensitivity and mechanical compliance, making it advantageous for detecting subtle deformations or large‐range body motion, the dense configuration remains more suitable for reliable and continuous biomechanical monitoring.

**FIGURE 4:**
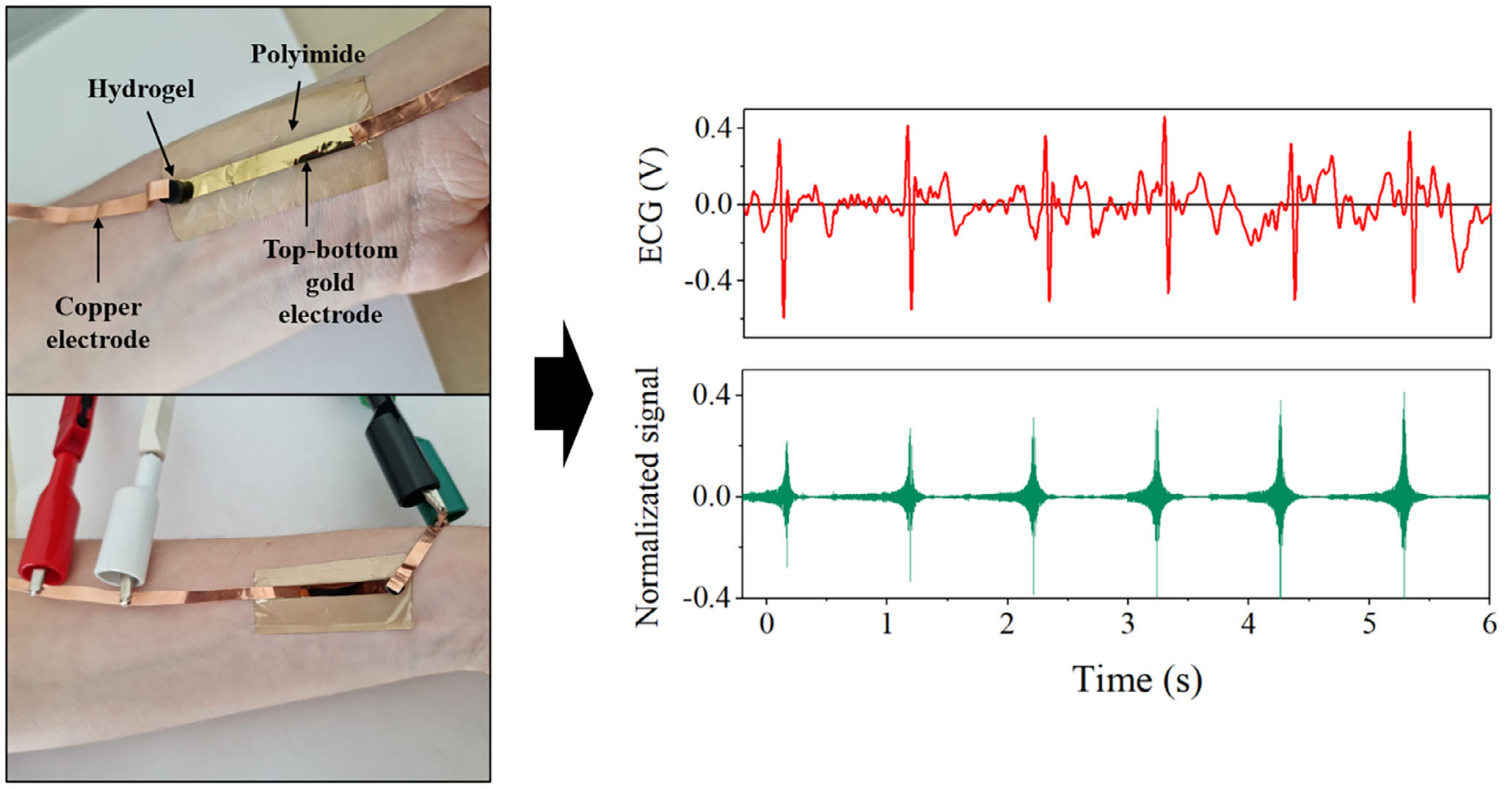
Physiological validation of the 3D‐printed dense cylindrical *f*‐1T‐MoS_2‐_ *_hyt/_* PEGDA/PANI hybrid hydrogel using an ultrathin flexible sensing electrode. Left: Photographs of the experimental setup using flexible ultrathin polyimide films with microfabricated Cr/Au electrodes integrated with the hybrid hydrogel and conformally placed over the radial artery at the wrist. Right: Simultaneously recorded ECG signal (top, red line) and ultraprocessed normalized signal obtained from the hydrogel (bottom, green line).

To demonstrate the practical applicability of the proposed material, the dense cylinder architecture was selected for wearable physiological measurements because it exhibited superior electromechanical performance and signal stability compared with the gyroid architecture. The hydrogel was integrated into an ultrathin flexible sensing platform based on polyimide films with microfabricated Cr/Au electrodes (Figure 4 left). The flexibility of the substrate enabled conformal contact with the skin over the radial artery, ensuring stable electromechanical measurements during pulse acquisition. The normalized hydrogel‐derived signal exhibited clear temporal synchronization with the simultaneously recorded ECG waveform, consistent with the behavior previously observed in the experimental set‐up (Figure 1C). In both cases, a slight delay between the ECG and the hydrogel‐derived signal was observed (Figure 4 right), which is attributed to the PTT, corresponding to the propagation of the arterial pressure wave from the heart to the peripheral artery [3, 82]. Although this behavior is consistent with PTT, a detailed investigation and quantitative analysis will be addressed in future studies. Furthermore, the sensor maintained a stable electrical response during a continuous acquisition period of 6 min (Figure S16), demonstrating its suitability for continuous physiological monitoring. These results demonstrate the feasibility of combining hybrid hydrogels with ultrathin flexible electronics for non‐invasive cardiovascular monitoring [83].

Compared with recently reported MoS_2_‐based hydrogel sensors (Table 2), the proposed platform uniquely integrates DLP 3D‐printed architectures, stable electromechanical performance within the physiological pressure range, and ECG‐correlated wearable pulse monitoring. The programmable dense and gyroid geometries enable tunable trade‐offs between pressure sensitivity, strain responsiveness, and signal stability, demonstrating the potential of architected hybrid hydrogels for next generation wearable cardiovascular sensing applications.

**TABLE 2:** Performance comparison of MoS_2_‐based hydrogel wearable pressure sensors reported in recent literature.

Sensor material	Fabrication	Pressure sensing	Sensitivity/GF	Wearable application	Ref.
2D MoS_2_/PEGDA/PANI covalent hydrogel	UV‐cured hydrogel	1.5 kPa of pressure and 30% of strain	Δ*I*/*I*_0_ ≈500 (at 1.5 kPa of pressure) and ≈1.8 µA (at 30% of strain)	Wearable strain/pressure sensing	[23]
DNH/MoS_2_NS/EG‐PEDOT: PSS hydrogel	Double‐network conductive hydrogel	Up to 500% strain	GF = 1.67	Real‐time motion tracking	[84]
Alginate/MoS_2_NS/AgNW hydrogel	3D‐printed wearable bioink	Dynamic touch/pressure sensing	Sensitivity of 0.15 kPa^−1^	Sweat and touch monitoring	[85]
MoS_2_‐PVA‐PAA‐Li^+^ conductive hydrogel	Conductive dual‐network hydrogel	Up to 780% tensile strain	GF = 1.17	Wearable strain sensing	[10]
Gel‐PEG‐MoS_2_ hydrogel	NIR light irradiation	Strain range of 30%–70%	GF = 1.13	Flexible electronic sensors	[86]
EG/MoS_2_‐PAAM‐Fe^3+^ organohydrogel	Chemical cross‐linked	Tensile strength of 8.81 MPa	Sensitivity of 0.06 kPa^−1^	Electronic materials	[87]
AAM/CNT/MoS_2_ hydrogel	UV‐cured hydrogel	50% of strain	6.1 nm for dopamine	Biosensors for dynamic neurochemical sensing	[88]

## 3 Conclusions

In summary, this study presents two types of 2D 1T‐MoS_2_ morphologies obtained following two different methods: a top‐down chemical exfoliation (1T‐MoS_2‐_ *_exf_*) and a bottom‐up hydrothermal synthesis using _L_‐Cysteine (1T‐MoS_2‐_ *_hyt_*). The two materials were then incorporated within the polymeric matrices of PEGDA and PEGDA/CH, and the resulting hydrogels were characterized in terms of electrical response and mechanical stability. The resulting hybrid hydrogels demonstrate a linear and reversible piezoresistive response in a pressure range compatible with that of peripheral blood under cyclic deformation.

Particularly, the systems based on *f*‐1T‐MoS_2‐_ *_exf_*/PEGDA/CH/PANI exhibited higher gauge factors (21.7), while *f*‐1T‐MoS_2‐_ *_hyt_*/PEGDA/CH/PANI (GF 9.1) provided greater mechanical strength, structural stability, and a reproducible signal response under few cycles loading. The incorporation of chitosan (CH) improved energy dissipation and mechanical stability, reducing hysteresis and increasing structural strength during cyclic deformation.

The optimized *f*‐1T‐MoS_2‐_ *_hyt_*/PEGDA/CH/PANI hydrogel demonstrated stable, reversible current responses under dynamic mechanical stimulation and was successfully applied for pulse monitoring at the wrist, yielding physiologically relevant heart rate values (∼85 bpm) with temporal correlation to ECG signals. Additionally, preliminary in vitro studies using MRC5 fibroblasts confirmed good biocompatibility, supporting the potential for wearable applications 3D printing via digital light processing enabled precise control over hydrogel architecture, revealing a clear trade‐off between sensitivity and mechanical stability. Dense cylindrical structures provided superior pressure sensitivity and signal stability within the physiological pressure range (≈10–16 kPa), whereas gyroid architectures exhibited higher strain sensitivity but increased signal variability due to their porous, deformation‐driven conductive network reconfiguration. Furthermore, the dense 3D‐printed architecture was successfully integrated into an ultrathin flexible sensing platform for physiological pulse monitoring. These findings highlight the critical role of architectural design in tuning electromechanical performance, thus the potential of combining conductive 2D nanomaterials with DLP‐enabled structural engineering strategies to develop miniaturized and customizable hydrogel‐based sensing platforms.

Future work will focus on extending 3D printing to chitosan (CH)‐based hydrogels. Although CH improves mechanical stability, energy dissipation, and reduces hysteresis in UV‐cured systems, its use in DLP printing is limited by its low photocurability. Overcoming these challenges will enable the fabrication of fully biocompatible and biodegradable 3D‐printed hydrogels while preserving their enhanced electromechanical performance, thereby advancing their application in wearable sensing.

## 4 Experimental Section

### 4.1 Materials

Molybdenum disulfide 2 µm powder (MoS_2_, 98%), p‐anisidine (99%), n‐butyllithium (2.5 m in dry hexane), sodium molybdate (NaMoO_4_, 98%), _L_‐Cysteine (97%), 4‐Methoxybenzenediazonium tetrafluoroborate (98%), potassium persulfate (≥99.0%), hydrochloric acid (30%), medium molecular weight chitosan (CH) (Mw = 190–310 kDa, deacetylation degree of 75–85%), methacrylic anhydride (MA, 94%), acetic acid (96%), Lithium phenyl‐2,4,6 trimethylbenzoylphosphinate (LAP, ≥95%), and polyethylene glycol diacrylate (Mw: 700), aniline (for synthesis), were purchased from Sigma–Aldrich and used without further purification. The water used was Milli‐Q grade, and hexane and ethanol were HPLC quality.

### 4.2 Chemical Exfoliation of MoS_2_ (1T‐MoS_2‐exf_)

The synthesis of 1T‐MoS_2_ was prepared following the chemical exfoliation procedure described by Domenici et al. [23]. Thereby, bulk MoS_2_ (1 g, 6.25 mmol) was left drying for 1 h under vacuum with mild heating (40°C) and stirring in a pre‐dried Schlenk line glassware. Then, the dried bulk material was stirred for 72 h in dry n‐butyl lithium (6 mL, 15 mmol) at room temperature. The resulting solution of Li_x_MoS_2_ was cooled to room temperature, and then hexane (5 × 30 mL) was used to remove organic impurities by centrifugation (7000 rpm for 12 min), and dried under vacuum for 1h. Afterward, the powder was dispersed in water (2 mg/mL) and sonicated using a tip sonicator for 2 h. Finally, the 1T‐MoS_2_ was centrifugated (6000 rpm for 20 min), and the supernatant was recovered and freeze‐dried to obtain 2D‐MoS_2_ powder.

### 4.3 Hydrothermal Synthesis of MoS_2_ (1T‐MoS_2‐hyt_)

The synthesis of MoS_2_ was developed from the one‐step hydrothermal synthesis proposed by Kasinathan et al. [89]. First, sodium molybdate (0.36 g, 1.74 mmol) and _L_‐Cysteine (0.72 g, 5.94 mmol) were dissolved in 40 mL of Milli‐Q water. The obtained solution was transferred into a 50 mL Teflon‐lined stainless‐steel autoclave and heated at 180°C for 24 h. After cooling down to room temperature, the material was collected by vacuum filtration and underwent several washing cycles with Milli‐Q water to remove the residual reactants; finally, it was dried under vacuum.

### 4.4 Preparation of UV‐Cured Hydrogels

PEGDA‐based hydrogels were produced by mixing 2 g of PEGDA, 0.2% of the photoinitiator LAP, and 10 mg of the prepared of *f*‐MoS*_2‐exf_* and *f*‐MoS*_2‐hyt_* (separately) into 10mL of water, obtaining two distinct hydrogel formulations. PEGDA/CH hydrogels were produced by mixing 2 g of PEGDA, 0.2% of the photoinitiator LAP, and 10 mg of the prepared of *f*‐MoS*_2‐exf_* and *f*‐MoS*_2‐hyt_* (separately) with 10 mL of CH solution (1.5 wt.% lyophilized CH in a 2 wt.% aqueous acetic acid solution prepared 12 h beforehand), resulting in two additional formulations.

The suspensions were then sonicated for 15 min (for the controls hydrogels that do not contain *f*‐MoS_2_, the same procedure was used). After this step, 1 mL of the formulations were poured into a cylindrical silicone mold (0.5 cm in diameter), irradiated for 1.5 min with a power density 100 mW/cm^2^ (correspondent to an energy 9000 mJ/cm^2^), using a Hamamatsu LC8 lamp with an 8 mm light guide and a spectral distribution range of 240–400 nm. Afterward, the obtained *f*‐MoS*_2‐exf_* and *f*‐MoS*_2‐hyt_* based on PEGDA and PEGDA/CH hydrogels were washed by immersion with Milli‐Q water for 20 min. The hydrogels measured approximately 0.5 cm in diameter and 0.5 cm in height.

### 4.5 Preparation of DLP Hydrogels

*f*‐MoS_2‐_ *_hyt_*/PEGDA formulation (2 g of PEGDA, 0.2% of the photoinitiator LAP, and 10 mg of the prepared of *f*‐MoS_2‐_ *_hyt_*) was additively shaped using a DLP‐based stereolithographic printer (Admaflex 130, Admatec Europe BV, The Netherlands). The photo crosslinking is due to a 405 nm UV LED projector, with a pixel size of 50 µm. This layer‐by‐layer photopolymerization approach enables the fabrication of miniaturized architectures with high geometric precision, making it particularly suitable for wearable sensing applications. The process achieves a spatial resolution of approximately 50 µm in the x–y plane (defined by the projector pixel size) and 50 µm in the z direction, determined by the set layer thickness, which can be further reduced to as low as ca. 10 µm. A customized vat set‐up was employed; lift and down speeds were set to 2 mm/min to limit stress on the samples photopolymerized on the moving aluminum platform. A delay before exposure of 0.5 s allowed the homogenous spreading of the ink and the removal of any possible air bubbles; however, these issues were already largely mitigated by the desirable liquid‐like viscosity of the slurry. Samples were exposed to UV radiation for 5 s at a power density of 20.2 mW/cm^2^ (energy 101 mJ/cm^2^). UV‐curing depth tests were systematically conducted using different exposure times and power densities to carefully optimize the DLP printing parameters and identify conditions providing both high printing fidelity and reliable interlayer adhesion. The selected parameters ensured a curing depth at least three times greater than the applied layer thickness (50 µm), while simultaneously minimizing overcuring and preserving the resolution of the printed features. Representative curing depth measurements, printed test patterns, and a summary of the investigated exposure conditions have been added to Figure S14. These parameters enabled good interlayer adhesion and high manufacturing resolution, allowing the precise manufacturing of two geometries: a dense cylinder and a gyroid cylinder with flat upper and lower surfaces, as shown in Figure 2 [37]. After printing, parts were rinsed with ethanol (3 × 100 mL) until performing characterizations or applicative measurements.

### 4.6 PANI Impregnation of UV‐Cured and DLP‐Printed Hydrogels

The confined‐space impregnation of PANI was carried out using the stepwise procedure described by Domenici et al. [23]. Initially, the target gels were immersed in a 1 m HCl solution containing 50 mg of K_2_S_2_O_8_ (0.18 mmol, 1.5 eq) for 2 h. Subsequently, the gels were transferred into a 0.025 m aniline solution prepared in hexane and left to react for 16 h. Finally, the *f*‐MoS_2_ *_‐exf_* and *f*‐MoS_2_ *_‐hyt_* based on PEGDA and PEGDA/CH hydrogels underwent a washing procedure, first by immersion in 1 m HCl for 1 h, followed by immersion in pure Milli‐Q water for an additional hour.

### 4.7 Characterization of Materials and Hydrogels

All prepared materials were characterized using TEM, zeta potential, XRD, RAMAN, TGA, FT‐IR, and XRD. A detailed description of the techniques and procedures adopted for materials characterization is reported in Section S1. The gravimetric approach was used to determine the swelling ratio of different hydrogels in Milli‐Q water at room temperature for 24 h. The equilibrium swelling ratio (ESR) was calculated using Equation (1) [90]:

(1) $$\%ERS=\frac{W_{s}-W_{d}}{W_{d}}\times 100$$

Where W_s_ is the weight of the swollen hydrogel, and W_d_ is the weight of the dried gel.

### 4.8 Electromechanical Characterization of Hydrogels and Pulse Detection

The electromechanical response of gels was characterized by cyclic compression tests on an Instron 3365 dynamometer equipped with a 10 N load cell, coupled with current measurements on a Keithley 2612B Sourcemeter. To evaluate both the gauge factor and a preliminary indication of the robustness of the measurements, displacement was applied at the rate of 2 mm/min until 20% strain and released until 50 mN, then the procedure was repeated for a total of 10 cycles for the hydrogels made using the lamp (UV‐cured) and 20 cycles for hydrogels made with the 3D printer (DLP‐printed). A constant voltage of 1 V was applied to the samples throughout the test using copper electrodes, and the corresponding current was recorded. For UV‐cured and 3D printing hydrogels, two parallel copper electrodes were placed on the top‐bottom surface of the sample, at a distance of ∼3 mm, and the in‐plane current was measured (Figure S15). From the coupled strain‐current measurements, the gauge factor (GF) was calculated using Equation (2):

(2) $$GF=\frac{\Delta R/R_{0}}{\varepsilon }$$

where the gauge factor is determined from the initial linear change in resistance (R_0_) at low strain levels and does not apply once the resistance change (ΔR) becomes nonlinear, and ɛ is the applied strain. After, the standard metric for evaluating the performance of strain‐sensing materials. Subsequently calculation of the resistance and normalization over the signal maximum, from the slope of the linear regime (strain 𝜀 < 0.05). Furthermore, the electromechanical test of the hydrogels was characterized by stepped compression tests on a potentiostat IVIUM CompactStat2.h equipped with the IviumSoft 4.1184 software. For UV‐cured hydrogels (*f*‐1T‐MoS_2‐_ *_hyt/_* PEGDA/CH/PANI) were sandwiched between two copper pieces for the measurement of polarization curves, which were obtained by linear scanning voltammetry (LSV) at a scanning range of −0.3 to 0.3 V. For the dense cylindrical hydrogels (*f*‐1T‐MoS_2‐_ *_hyt/_* PEGDA/PANI) made with the 3D printer, the same electrical measurement protocol was used for the ultrathin and flexible polyimide‐based electrodes, where the microfabricated Cr/Au electrodes were connected to the potentiostat through two external copper electrodes. Pulse waveforms were acquired using a piezoresistive hydrogel sensor placed over the radial artery at the subject's wrist. Electrical measurements were performed using an IVIUM CompactStat2 potentiostat controlled via IviumSoft version 4.1184. The sensing interface consisted of a copper electrode–hydrogel–copper electrode configuration, mechanically stabilized with Kapton tape to ensure consistent contact during measurements (Figure 1A). Chronoamperometric recordings were conducted by applying a constant potential of 50 mV. Signals were collected over 180 s with a sampling interval of 0.003 s. The ECG signals were recorded using the ST1VAFE3BX biosensor (STMicroelectronics), with a sampling frequency of 800 Hz.

Signal processing was implemented in MATLAB following established pulse waveform analysis methodologies [91]. The raw signal was detrended to remove baseline drift and subsequently filtered using a fourth‐order Butterworth band‐pass filter (0.7–10 Hz) to isolate the physiological frequency range of arterial pulsation while suppressing low‐frequency drift and high‐frequency noise. The filtered signal was further smoothed using a Savitzky–Golay filter to reduce residual noise while preserving waveform morphology.

Systolic peaks were identified using a peak detection algorithm with two constraints: (i) a minimum peak‐to‐peak interval of 0.6 s, corresponding to a maximum detectable heart rate of approximately 100 bpm, and (ii) a minimum amplitude threshold defined as Equation (3):

(3) $$H_{\min}=\mu +0.1\sigma$$

where μ and σ denote the mean and standard deviation of the signal, respectively. This empirically defined threshold enabled the rejection of low‐amplitude fluctuations associated with noise while retaining physiologically relevant pulse features.

### 4.9 MTS Hydrogel Cytotoxicity Assay Against MRC5 Cells

The human fibroblast cell line MRC‐5 was cultured in Opti‐MEM Reduced Serum Medium (Gibco) supplemented with 3% fetal bovine serum (FBS). Cells were maintained at 37°C in a humidified atmosphere with 5% CO_2_ for MTS cell viability and ROS assays. Then, 5 × 10^4^ cells were seeded into each well of a 24‐well plate (24‐well Clear TC‐treated Multiple Well Plates, Corning Costar). Three replicates were included for each sample. A positive control sample and a negative control sample were also included. Subsequently, the hydrogels were placed on top of the cell monolayer in each of the three wells corresponding to the hydrogel samples, and 10% Triton X‐100 was added to the positive control wells. Samples were incubated at 37°C for 24 and 72 h. After incubation, the hydrogels were removed. Then, MTS solution (CellTiter 96 Aqueous One Solution Cell Proliferation Assay, Promega) was added to each well, and samples were incubated at 37°C for 1 h, protected from light. Subsequently, three technical replicates were obtained from each well of the 24‐well plate by transferring samples to a 96‐well plate (Corning 96‐well Clear Polystyrene Microplate, Corning Costar). An additional blank sample consisting of Opti‐MEM medium was included with three replicates. Finally, absorbance was measured at 492 nm.

### 4.10 Preparation of Ultrathin Flexible Polyimide Substrates

Glass slides with dimensions of 50 mm × 50 mm × 1 mm (D263T eco glass, SCHOTT AG, Mainz, Germany) were used as carrier substrates. The glass substrates were washed in a Miele professional washer (Miele & Cie. KG, Gütersloh, Germany) and dried with nitrogen. Next, the glass samples were treated with oxygen plasma for 5 min at 200 W power, and substrates were immersed into DI for 2 min. A mixture of phenylsilane 97% 300 ug (Sigma–Aldrich Chemie GmbH, Taufkirchen, Germany), 1H, 1H, 2H, 2H‐Perfluorodecyltriethoxysilane 97% 2150 ug (Alfa Aesar GmbH & Co. KG, Karlsruhe, Germany), and n‐hexane 500 mL (Fisher Scientific GmbH, Schwerte, Germany) was prepared, with n‐hexane serving as the solvent for both silanes. The treated glass substrates were immersed in the mixture for 20 min at room temperature, dried with nitrogen, and baked at 160°C for 2 min. The polyimide (PI) was prepared as described in our previous work [92]. After cooling, a polyimide precursor solution was spin‐coated at 3000 rpm/500 rpm/180 s onto the glass substrates three times to achieve a PI film thickness of approximately 3 µm (Figure S17B). After each spin‐coating step, the sample was placed on a hotplate at 220°C for 10 min to cure the PI film. Finally, the free‐standing polymer foil was obtained by releasing the spin‐coated film.

### 4.11 Fabrication of Ultrathin Flexible Electrodes on Polyimide Substrates

The electrodes were fabricated on the polymer foil while still attached to the glass handling substrates. The electrodes were patterned by a lift‐off technique using AZ5214E photoresist (Microchemicals GmbH, Ulm, Germany), which was optically patterned. A Cr^10nm^/Au^50nm^ bilayer was then deposited at room temperature under a pressure of 1.5 × 10^−7^ mbar. The lift‐off process was performed using acetone and isopropanol, with a final rinse in DI water. Ultimately, flexible, independent electronic devices were obtained by releasing the polymer film containing the fabricated contacts, resulting in flexible electronic devices with dimensions of 4.7 cm × 2.2 cm (Figure S17A) and a PI film thickness of ∼3 µm (Figure S17B).

### 4.12 ROS Level of MRC5 Cells Against Hydrogels

ROS level of MRC5 cells against hydrogels was assessed using the ROS‐Glo H_2_O_2_ Assay (Promega). 3 × 10^5^ cells were seeded into each well of a 24‐well plate (24‐well Clear TC‐treated Multiple Well Plates, Corning Costar). Three replicates were included for each sample. A negative control sample was also included. Thus, hydrogels were placed on top of the cell monolayer in each of the three wells corresponding to the hydrogel samples. Samples were incubated at 37°C for 24 and 72 h. After incubation, the hydrogels were removed, and the ROS measurement was performed by adding the H_2_O_2_ Substrate Solution to each well. The samples were incubated at 37°C for 2 h, protected from light. Subsequently, three technical replicates were obtained from each well of the 24‐well plate by transferring samples to a 96‐well plate (Corning 96‐well White Flat Polystyrene Microplate, Corning Costar). Finally, ROS‐Glo detection Solution was added to each well, and the well plate was incubated for 20 min at RT. ROS level was measured by reading the luminescence of the samples.

## Author Contributions

**Mattia Bramini**: investigation, resources, supervision, data curation, Writing – review and editing, validation, methodology. **Teresa Gatti**: conceptualization, funding acquisition, writing – review and editing, project administration, supervision, resources, investigation. **Luca Ceseracciu**: methodology, validation, visualization, writing – original draft, writing – review and editing, formal analysis, data curation, investigation. **Daniel Jiménez‐boland**: investigation, methodology, validation, data curation. **Domenico Galdiero**: investigation, resources, data curation, formal analysis, validation. **Sara Domenici**: investigation, methodology, data curation, validation, writing – original draft. **Micaela Pozzati**: investigation, data curation. **Erica Scarcelli**: investigation, writing – original draft, formal analysis, data curation, validation. **Jenny Flores**: conceptualization, investigation, methodology, validation, visualization, writing – original draft, writing – review and editing, formal analysis, project administration, data curation. **Paola Sánchez‐moreno**: resources, supervision, data curation, validation, writing – review and editing. **Marco Sangermano**: investigation. **Ignazio Roppolo**: validation, visualization, writing – review and editing. **Daniil Karnaushenko**: methodology, validation, writing – review and editing, supervision, resources. **Bartolomeo Coppola**: investigation, resources, supervision, data curation, formal analysis, validation, visualization, writing – original draft, writing – review and editing. **Rossella Sesia**: investigation. **Arianna Bertero**: investigation, writing – original draft, methodology, validation, visualization, formal analysis, data curation. **Nicolò Petrini**: investigation. **Mattia Spedicati**: investigation, validation, data curation. **Pavel Fedorov**: investigation, formal analysis.

## Conflicts of Interest

The authors declare no conflicts of interest.

## Supporting information




**Supporting File**: advs77092‐sup‐0001‐SuppMat.docx.

## Data Availability

The data that support the findings of this study are available from the corresponding author upon reasonable request.
