# Supplementary material for: Printable Piezoresistive Hybrid Hydrogels Based on 2D MoS2 for Flexible Pulse Sensing
Source: Adv Sci (Weinh). 2026 Aug 27:e77092. Online ahead of print. doi: 10.1002/advs.77092 (PMC13521617; doi:10.1002/advs.77092)
Supplement: Supplementary file 1 [file ADVS-9999-e77092-s001.docx]

Supporting Information for:

Printable piezoresistive hybrid hydrogels based on 2D MoS_2_ for flexible pulse sensing.

Jenny Flores, Arianna Bertero, Luca Ceseracciu, Daniel Jiménez-Boland, Sara Domenici, Mattia Bramini, Paola Sánchez-Moreno, Erica Scarcelli, Domenico Galdiero, Pavel Fedorov, Daniil Karnaushenko, Rossella Sesia, Ignazio Roppolo, Micaela Pozzati, Mattia Spedicati, Nicolo Petrini, Marco Sangermano, Bartolomeo Coppola, Teresa Gatti

**Experimental details and characterization**

*Functionalization with diazonium salts*

The MoS_2_ was functionalized according to the procedure already described by Beanson *et al.*^[1]^ Initially, 100 mg of 1T MoS_2_ (MoS_2_**_-_*_e_****_xf_* and MoS_2_**_-_***_hyt_*) were suspended in 100 ml of Milli-Qwater and then 5 mL of a 10 mg/mL solution of 4-Methoxybenzenediazonium tetrafluoroborate were added dropwise (98%). The solution was then allowed to stir overnight. The resulting *f-*MoS_2_**_-_*_e_****_xf_* and *f*-MoS_2_**_-_***_hyt_* functionalized material were collected by ﬁltration and washed several times with water (5×100 ml) to remove any unreacted material, then dried under vacuum.

*Synthesis of methacrylated chitosan (MCH)*

The chitosan (CH) methacrylation was prepared following the procedure described by Sesia *et al*.^[2]^ First, the CH (1.5 wt%) was dissolved in acetic acid (2 wt%) solution and stirred vigorously until homogenization. Subsequently, methacrylic anhydride (MA) was added dropwise maintaining a ratio of 1:20 between the aminoglucose moieties and MA. The reaction was performed for 4 hours at 50 °C under constant stirring. The resulting solution was dialyzed against distilled water for 4 days using a cellulose dialysis membrane with a molecular weight cut-off (MWCO) of 14 kDa and then freeze-dried to recover the methacrylated chitosan (MCH).

**Characterization**

*High-resolution transmission electron microscopy*

Size and morphology of the 1T-MoS_2-_*_exf_* and 1T-MoS_2_**_-_***_hyt_* were obtained from h*igh-resolution* transmission electron microscopy (HR-TEM). HR-TEM experiments were performed on a TALOS F200X (Thermo Fisher Scientific, Eindhoven, Netherlands) system operated at an accelerating voltage of 200 kV.

*Zeta potential*

The zeta (ζ) potential on the surface of MoS_2-_*_exf_* and MoS_2-_*_hyt_* was measured using an electrokinetic analyzer (Litesizer DLS 500 Anton Paar) equipped with an automatic titration unit. 2 mg of MoS_2-_*_exf_* and MoS_2-_*_hyt_* powder were dispersed in 3 ml of Milli-Q water and sonicated for 15 min. Fifty points were measured for each number of runs and each measurement was performed in triplicates.

*X-Ray diffraction (XRD)*

The structure of the and the amorphous phase characteristic of the 1T-MoS_2_ (MoS_2_**_-_*_e_****_xf_* and MoS_2_**_-_***_hyt_*) was determined by XRD. Experiments were made in the Bragg-Bretano geometry using a PANalytical X’Pert diffractometer with Cu Kα (λ1 = 1.54187 Å with a step size of 0.026 2θ), and a tube voltage and current of 40 kV and 40 mA, respectively.

*Raman spectroscopy*

Raman spectroscopy was used to observe the change in crystallinity of MoS_2_. Raman spectra were collected at room temperature with a Renishaw InVia spectrometer, using a 532 nm laser and using 50 times magnification. The spectra were collected using the following instrumental settings: 6 s of integration time, 60 co-addition and a laser power of 0.1 mW.

*Thermogravimetric analysis*

Thermogravimetric analysis was employed to determine the amount of functionalization (*f*-MoS_2_(–Ph–OCH_3_)) of MoS_2_ and was performed using a Netzsch STA409PC instrument. The measurements were performed under air starting from 25 °C to 900 °C with ramp of 10 °C/min^-1^. The functionalization degree (FD), i.e. the number of MoS_2_ molecules per functional group (–Ph –OCH_3_), was obtained for all functionalized samples using Equation (S1).^[3]^

$FD=\frac{R \left( \% \right)\cdot M_{w}(g/{mol})}{L \left( \% \right)\cdot160.07 (g/{mol})}$	(S1)

In this equation (Equation S1), R and L represent the residue (%) and weight loss (%) measured at 650 °C, respectively, which is attributed to the degradation of the functional groups. ^[4]^ The anticipated functional group attached to the MoS_2_ surface is expressed as (*M*_w_), and the molecular weight of MoS_2_ was calculated to be 160.07 g/mol.

*FT-IR spectroscopy*

To conﬁrm the successful functionalization (*f*-MoS_2_(–Ph–OCH_3_)) of MoS_2_, attenuated total reﬂectance-infrared spectroscopy (ATR-FTIR) was used. The experiments were performed on air using a Thermo Scientiﬁc Nicolet iS50 FTIR spectrometer (Thermo Fisher Scientiﬁc, Milano, Italy) equipped with a diamond crystal ATR accessory. ATR spectra were collected with a resolution of 4 cm^−1^ in the range of 4000–600 cm^−1^.

**Supporting Figures**

**Figure S1**. TEM imagens of A-B) 1T-MoS_2-_*_exf_* nanoflakes*_,_* C-D) 1T-MoS_2-_*_hyt_* nanosheets*_._* Inset magniﬁcations of HR-TEM images of the multi-layers of B) 1T-MoS_2-_*_exf_* nanoflakes and D) 1T-MoS_2-_*_hyt_* nanosheets.

**Figure S2**. Zeta potential distribution of materials A) 1T-MoS_2-_*_exf_* and B) 1T-MoS_2-_*_hyt_*.

**Figure S3**. XRD spectra of the synthesized of 1T-MoS_2-_*_exf_* (black) and 1T-MoS_2-_*_hyt_* (violet).

**Figure S4**. Raman spectrum of the A) synthesized 2H-bulk form (red), 1T-MoS_2-_*_exf_* (black) and 1T-MoS_2-_*_hyt_* (violet), and B) functionalized materials of *f*-1T-MoS_2-_*_exf_* (blue) and *f*-1T-MoS_2-_*_hyt_* (red). The spectra are taken with an excitation wavelength of 532 nm. C) FT-IR spectra of the synthesized of 1T-MoS_2-_*_exf_* (black), *f*-1T-MoS_2-_*_exf_* (blue), 1T-MoS_2-_*_hyt_* (violet) and *f*-1T-MoS_2-_*_hyt_* (red).

**Figure S5**. Thermogravimetric weight loss comparison between functionalized material with –Ph–OCH_3_ (*f*-MoS_2-_*_exf_* (green) and *f*-MoS_2-_*_hyt_* (violet) and 1T-MoS_2_ (MoS_2-_*_exf_* (black), and MoS_2-_*_exf_* (orange).

**Table S1.** Functionalization degree of *f*-1T-MoS_2_*_-exf_* and *f*-1T-MoS_2_*_-hyt_* by TGA.

| Sample | Weight lost (%) ^a^ | FD ^b^ |
| --- | --- | --- |
| *f*-1T-MoS_2-_*_exf_* | 41.80 | 1.6 |
| 1T-MoS_2-_*_exf_* | 18.76 | - |
| *f*-1T-MoS_2-_*_hyt_* | 41.85 | 2.0 |
| 1T-MoS_2-_*_hyt_* | 22.15 | - |

^a)^ Determined weight loss at 650 °C.

^b)^ Number of MoS_2_ molecules per functional group.

**Figure S6**. The equilibrium swelling ratio (ESR) over time of the hydrogels of *f*-1T-MoS_₂-_*_exf_*/PEGDA/PANI (blue), *f*-1T-MoS_₂-_*_exf_/*PEGDA/CH/PANI (green), *f*-1T-MoS_₂-_*_hyt_*/PEGDA/PANI (gray) and *f*-1T-MoS_₂-_*_hyt_*/PEGDA/CH/PANI (red).

**Figure S7.** The equilibrium swelling ratio (ESR) over time of the DLP-printed hydrogels of *f*-1T-MoS_₂-_*_exf_*/PEGDA/PANI: A) Dense cylinders (blue), B) Gyroid cylinders (red).

**Figure S8.** Mechanical hysteresis of piezoresistive hybrid hydrogel based on A) *f-*1T-MoS₂*_-exf_*/PEGDA/CH/PANI (green) and C) *f-*1T-MoS₂*_-exf_*/PEGDA/PANI (blue). Electromechanical response of hybrid hydrogels B) *f-*1T-MoS₂*_-exf_*/PEGDA/CH/PANI (orange) and D) *f-*1T-MoS₂*_-exf_*/PEGDA/PANI (brown). Insets show the electrical output performance of hydrogels for 10 cycles of continuous pressure.

**Figure S9**. Mechanical hysteresis of piezoresistive hybrid hydrogels based on A) *f*-1T-MoS₂*_-hyt_*/PEGDA/CH/PANI (red) and C) *f*-1T-MoS₂*_-hyt_*/PEGDA/PANI (gray). Electromechanical response of hybrid hydrogels B) *f*-1T-MoS₂*_-hyt_*/PEGDA/CH/PANI (purple) and D) *f*-1T-MoS₂*_-hyt_*/PEGDA/PANI (brown). Insets show the electrical output performance of hydrogels for 10 cycles of continuous pressure.

**Figure** **S10**. Evaluation of the piezoresistive behavior of hybrid hydrogels. A) The I-V curves of the *f*-1T-MoS_2-_*_exf_* /PEGDA/CH/PANI, B) *f*-1T-MoS_2-_*_hyt_*/PEGDA/CH/PANI and C) resistance trends of *f*-1T-MoS_2-_*_exf_*/PEGDA/CH/PANI and D) *f*-1T-MoS_2-_*_hyt_*/PEGDA/CH/PANI free-standing hydrogels as a function of the applied pressure on the top of the hydrogel surface between two copper pieces.

**Figure S11**. Evaluation of the piezoresistive behavior of hybrid hydrogels. A) The I-V curves of the *f*-1T-MoS_2-_*_exf_* /PEGDA/PANI, B) *f*-1T-MoS_2-_*_hyt_*/PEGDA/PANI and C) resistance trends of *f*-1T-MoS_2-_*_exf_*/PEGDA/PANI and D) *f*-1T-MoS_2-_*_hyt_*/PEGDA/PANI free-standing hydrogels as a function of the applied pressure on the top of the hydrogel surface between two copper pieces.

**Figure S12**. Evaluation of the piezoresistive behavior of hybrid hydrogels. **A)** The I-V curves of the PEGDA/PANI, **B)** PEGDA/CH and **C)** resistance trends of PEGDA/PANI and **D)** PEGDA/CH/PANI free-standing hydrogels as a function of the applied pressure on the top of the hydrogel surface between two copper pieces.

**A)**

**B)**

**Figure S13**. The resting pulse response located on the wrist by: A) unprocessed chronoamperometric current response of the *f-*1T-MoS₂*_-hyt_*/PEGDA/CH/PANI hydrogel for 30 seconds, and B) simultaneous acquisition of electrocardiography (ECG) waveform obtained from the ECG monitoring system (black line) and pulse from chronoamperometric current response of the *f-*1T-MoS₂*_-hyt_*/PEGDA/CH/PANI hydrogel (red line).

**Figure S14.** UV-curing depth tests performed prior to DLP printing using different exposure conditions. Chessboard patterns fabricated at decreasing UV power densities and exposure energies are shown together with the corresponding curing depth values. The condition selected for DLP fabrication (5 s exposure at 20.2 mW/cm², corresponding to 101 mJ/cm²) provided the best compromise between printing resolution, edge definition, and curing depth.

**Figure S15**. Schematic representation of the electromechanical measurement of hydrogels. Compression test on a hydrogel with the electrodes at the top-bottom.

**Figure S16**. Normalized chronoamperometric response of the dense cylindrical 3D-printed *f*-1T-MoS_2-_*_hyt_*/PEGDA/PANI hydrogel during continuous wrist pulse monitoring over a 6 min acquisition period.

**Figure S17**. Ultrathin flexible electrodes on polyimide substrates fabricated for physiological monitoring. A) Optical image of the complete sensing platform with microfabricated Cr/Au electrodes and overall dimensions (4.7×2.2 cm). B) Photograph of the flexible polyimide-based sensing platform highlighting its ultrathin thickness (~3 μm).

**References**

[1]	E. E. Benson, H. Zhang, S. A. Schuman, S. U. Nanayakkara, N. D. Bronstein, S. Ferrere, J. L. Blackburn, E. M. Miller, *J. Am. Chem. Soc.* **2018**, *140*, 441.

[2]	R. Sesia, S. Ferraris, M. Sangermano, S. Spriano, *Polymers (Basel).* **2022**, *14*, 4645.

[3]	E. Er, H.-L. Hou, A. Criado, J. Langer, M. Möller, N. Erk, L. M. Liz-Marzán, M. Prato, *Chemistry of Materials* **2019**, *31*, 5725.

[4]	L. Hostert, M. S. Dias, C. B. de Aquino, F. C. dos Santos, V. S. Marangoni, C. de Carvalho C Silva, L. Seixas, C. M. Maroneze, *The Journal of Physical Chemistry C* **2024**, *128*, 20856.
